# Synthetic artificial intelligence in cardiology: from generative models to clinical applications

**DOI:** 10.1093/ehjopen/oeag026

**Published:** 2026-03-01

**Authors:** Gianmarco Parise, Roberto Ceravolo, Fabiana Lucà, Michele Massimo Gulizia, Cecilia Tetta, Orlando Parise, Federico Nardi, Massimo Grimaldi, Sandro Gelsomino

**Affiliations:** Carim School for Vascular Disease, Synthetic Artificial Intelligence with focus on Cardiovascular Medicine, Maastricht University, Universiteitssingel 50, 6222 ER, Maastricht, The Netherlands; Department of Cardiology, Lamezia Terme Hospital, Via Senatore Arturo Perugini 1, 88046, Lamezia Terme Lamezia, Italy; Carim School for Vascular Disease, MRI-based Artificial Intelligence for Cardiovascular Imaging, Maastricht University, Universiteitssingel 50, 6222 ER, Maastricht, The Netherlands; Department of Cardiology, Garibaldi-Nesima Hospital, Piazza Santa Maria di Gesú 5, 95124, Catania, Italy; Expert in Cardiovascular Imaging and Artificial Intelligence, Via Ruggi 14, 40137, Bologna, Italy; Synthetic Artificial Intelligence in Medicine, Department of Engineering, Universita della Calabria, Via Pietro Bucci, 87 036, Arcavacata, Cosenza, Italy; Department of Cardiology, S. Spirito Hospital, Via Giovanni Giolitti 2, 15033, Casale Monferrato, Alessandria, Italy; Department of Cardiology, Acqua Viva Delle Fonti Hospital, VStrada Provinciale 27 Acquaviva Santeramo-km 4, 70021, Acqua Viva Delle Fonti, Bari, Italy; Carim School for Vascular Disease, Synthetic Artificial Intelligence with focus on Cardiovascular Medicine, Maastricht University, Universiteitssingel 50, 6222 ER, Maastricht, The Netherlands

**Keywords:** Synthetic Artificial Intelligence, Cardiovascular Data Simulation, Generative Models in Medicine, Digital Twins and Synthetic Cohorts, AEthical and Regulatory Challenges in AI

## Abstract

Synthetic artificial intelligence (AI) is rapidly redefining biomedical research—yet in cardiovascular medicine, its clinical relevance remains obscure, underexplored, and underestimated. Unlike traditional AI, which interprets data, synthetic AI generates entirely new, patient-like information: from realistic ECG signals to cardiac imaging and virtual cohorts that simulate disease progression. While recent publications have addressed specific synthetic AI tools in cardiology, no prior review has comprehensively synthesized their architectures, clinical applications, and implementation challenges within a single, practice-oriented framework. This State-of-the-Art Review fills that gap. We provide a clear, critical synthesis of core architectures—Generative Adversarial Networks (GANs), Variational Autoencoders (VAEs), Diffusion Models, Transformers, Autoregressive Models, Digital Twins, and Synthetic Cohort Simulators—and map their emerging cardiovascular applications. We examine technical barriers, ethical concerns, regulatory uncertainties, and integration challenges, while anchoring the discussion in real-world clinical priorities. This review is not only a scientific analysis—it is a call to engagement. For academic researchers, it offers conceptual and technical clarity. For clinicians in resource-constrained settings, it presents synthetic AI not as abstract innovation, but as a practical opportunity to enhance diagnostic precision, optimize workflows, and extend clinical insight. Traditional AI supports cardiologists by interpreting data; synthetic AI extends this paradigm by creating new, clinically coherent information that enhances decision-making without replacing physician expertise. As investment grows and methods mature, cardiologists must shape this evolution—not as passive adopters, but as active drivers. This review invites them to take the wheel.

## Introduction

Artificial intelligence (AI) has increasingly reshaped cardiovascular medicine over the past decade, supporting image interpretation,^1,2^ enhancing risk prediction,^3–5^ and assisting clinical decision-making.^6,7^ Alongside these analytical tools, a distinct class of AI has recently emerged-synthetic or generative in nature. Unlike conventional models that focus on classification or prediction, synthetic AI systems are designed to create new data. Synthetic Artificial Intelligence refers to AI systems capable of generating novel, realistic, and clinically coherent data-such as images, waveforms, anatomical models, or virtual patient records-by learning the underlying distributions of real clinical datasets. It functions as an umbrella term that includes generative AI models (e.g. GANs, VAEs, diffusion models), but also extends to simulation-based and physiology-informed frameworks such as digital twins and synthetic cohort simulators. In this Review, the term ‘synthetic AI’ is therefore used in a broader sense than ‘generative AI,’ encompassing all methods that produce new patient-like information rather than merely analyzing existing data. These technologies enable the creation of synthetic cardiac images for model training, realistic ECG waveforms for arrhythmia classification, whole-heart anatomical reconstructions, and dynamic simulations of patient-specific physiology. As such, synthetic AI has the potential to support research, augment datasets, reduce privacy constraints, and complement clinical workflows across imaging, electrophysiology, interventional planning, and risk modelling. This Review summarizes the foundations of synthetic AI, describes the principal model families involved, and outlines emerging cardiovascular applications, highlighting opportunities, current limitations, and considerations for clinical translation.

## Overview of synthetic AI methods

Synthetic AI models can be broadly categorized by their generative mechanisms. While their architectures differ, these systems share a common purpose: Producing synthetic data that resembles real clinical information. This section provides a high-level overview of the most relevant methods in cardiovascular science. For technical readers or those seeking details on model architecture and training logic, we refer to the Supplementary Material (Supplementary material online, *Figures S1*-*S7*), which provide detailed visual explanations. All clinical images used in this Review were selected from anonymized cases contained in the institutional imaging archive of the Radiology Department, Reggio Calabria Hospital.

### Generative adversarial networks

Generative adversarial networks (GANs) are machine learning models that learn to produce realistic synthetic data-such as ECGs, cardiac images, or tabular patient profiles-by pitting two neural networks (a generator and a discriminator) against each other in an adversarial loop.^8,9^ Originally introduced by Goodfellow *et al*.^10^ this framework enables high-quality data generation without needing labelled datasets.^11,12^ GANs have evolved into specialized forms such as CGAN, DCGAN, and CTGAN, which are particularly effective with structured medical data.^13–19^

### Variational autoencoders

Variational autoencoders (VAEs) compress clinical data into a compact latent space and reconstruct outputs that retain core physiological features.^20,21^ Unlike traditional autoencoders, VAEs are probabilistic and capable of generating multiple variations of input data. Their stability and interpretability make them well-suited for modelling anatomy, signals, and patient phenotypes, especially when data are incomplete or limited.

### Transformers

Originally developed for language processing, Transformers have become central to synthetic AI due to their ability to model long-range dependencies using self-attention.^22^ This allows simultaneous analysis of complex temporal and structured inputs, such as sequential ECG waveforms, longitudinal clinical records, or integrated imaging-text data.^23–25^

### Autoregressive models

Autoregressive models predict the next element in a sequence based on previous values.^26^ When combined with Transformer backbones, they generate coherent synthetic narratives or time-series data.^27,28^ These models are commonly used in AI tools for event forecasting, clinical documentation, and temporal signal modelling.

### Diffusion models

Diffusion models generate high-resolution synthetic data by learning to reverse a noise process applied to real inputs.^29^ Unlike GANs, they are not adversarial but rely on probabilistic denoising to produce photorealistic and anatomically plausible outputs. They are emerging as a preferred solution for generating detailed cardiac imaging and waveform reconstructions.

### Digital twins

A digital twin is a virtual model of a patient or physiological system that updates dynamically using multimodal real-world data.^30–33^ This allows physicians to simulate treatments, anticipate outcomes, and plan procedures in a non-invasive, patient-specific way. Digital twins integrate imaging, labs, EHRs, and sensor inputs into adaptive simulations that mirror the evolving clinical state.

### Synthetic cohort simulators

These systems generate virtual populations that statistically mirror real-world cohorts.^34^ They are useful for privacy-preserving research, regulatory simulations, and machine learning training. Unlike digital twins, which are patient-specific and dynamic, synthetic cohorts model population-level data distributions and disease patterns at scale.

A summary of the core characteristics of the main families of synthetic AI methods described in this section is provided in *Table 1*.

**Table 1:** Key characteristics of synthetic AI methods

Method	Core mechanism	Key features
GANs	Adversarial training (generator vs. discriminator)	High-fidelity synthesis, good for images and ECG augmentation
VAEs	Probabilistic latent encoding/decoding	Interpretable latent space, multimodal fusion
Transformers	Self-attention on sequences	Long-range modelling for ECG/EHR, multimodal integration
Autoregressive models	Sequential prediction of next value	Transparent, physiologic time-series modelling
Diffusion models	Progressive denoising from noise to signal	High-resolution synthesis, stable training
Digital twins	Mechanistic + data-driven simulation	Patient-specific dynamic simulation
Synthetic cohorts	Population-level distribution modelling	Privacy-preserving virtual populations

## Applications of synthetic methods in Cardiology

*Table 2* provides an overview of representative cardiovascular applications of synthetic AI across imaging, electrophysiology, risk modelling, and simulation, together with key advantages and limitations relevant to clinical translation.

**Table 2:** Cardiovascular applications of synthetic AI: use cases, advantages, and limitations

Application	Method(s)	Advantages	Limitations
Border detection in poor echo windows	GANs, Transformers	Improved segmentation, artefact correction	May hallucinate borders; needs validation
Scar segmentation (CMR-LGE)	GANs, VAEs	High-fidelity scar modelling, augmentation	Potential artefacts, regulatory hurdles
ECG augmentation for arrhythmias	GANs, Diffusion, VAEs	Balances rare classes, improves classifier accuracy	Risk of unrealistic morphologies
Virtual patient cohorts	Synthetic cohorts, GANs	Privacy-preserving, scalable datasets	Limited provenance, potential bias
Digital-twin simulation for EP	Digital Twins	Patient-specific therapy planning	High computational demand
Perfusion/ischaemia anomaly detection	VAEs, Diffusion	Unsupervised detection, reduced labelling	Lower fidelity than supervised models

### Generative adversarial networks

**Figure 1:**
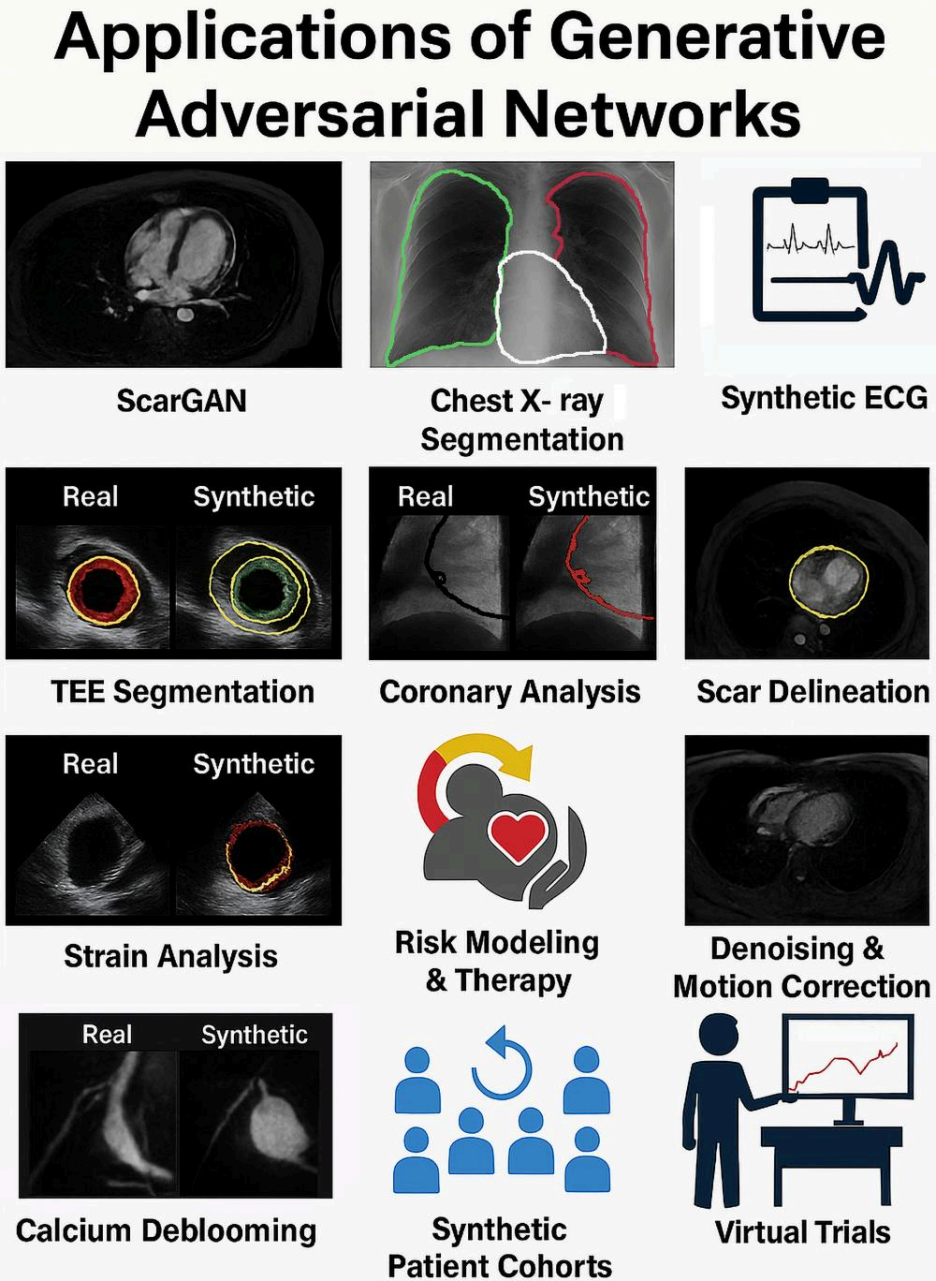
Applications of generative adversarial networks in cardiology. Overview of generative adversarial network applications in cardiology. Panels illustrate a range of use cases, including synthetic image generation (e.g. myocardial scar via ScarGAN), segmentation in chest X-ray and transoesophageal echocardiography, synthetic ECG waveform creation, coronary analysis, myocardial strain mapping, denoising, motion correction, calcium deblooming, synthetic patient cohort simulation, and virtual clinical trials. For architectural and functional details, see Supplementary Material (Sections  *S1*–S2). ECG, Electrocardiogram; GAN, Generative Adversarial Network; ScarGAN, Scar Generative Adversarial Network; TEE, transoesophageal Echocardiography. All clinical imaging examples (echocardiography, MRI, CT, and X-ray) shown in this figure originate from anonymized real patient data obtained from the authors’ institutions and used under institutional permission.

As shown in *Figure 1*, GANs are being actively explored across several cardiovascular domains. Their ability to generate high-fidelity synthetic data makes them valuable for augmenting limited datasets, enhancing image quality, and simulating clinical scenarios.

In imaging, GANs have been used to simulate myocardial scar patterns in cardiac MRI (ScarGAN), improving segmentation accuracy in patients with structural heart disease.^35^ Similar methods have stabilized cardiac boundary detection on chest X-rays.^36^ In electrocardiography, synthetic ECG signals produced by GANs have helped train and balance arrhythmia detection models, especially in underrepresented classes.^37^

Beyond image and signal augmentation, GANs support advanced modelling of patient trajectories and disease progression.^38^ Applications have included segmentation of the left atrial appendage on transoesophageal echocardiography (TEE),^39^ coronary flow analysis from intravascular ultrasound,^40^ and scar delineation in cardiac MRI without contrast agents.^41^ Emerging studies have demonstrated their potential in automating myocardial strain analysis, performing comparably to conventional speckle-tracking methods while reducing processing time.^42^

In cardiometabolic applications, GAN-powered simulations have improved personalized risk prediction and therapy modelling. In nuclear cardiology, GANs have been applied to reduce noise and correct motion artefacts in myocardial perfusion imaging,^43,44^ and semantic-oriented GANs have improved specificity in coronary CT angiography by minimizing calcium blooming artefacts.^45^

Finally, GANs are being explored as tools to create entirely synthetic patient cohorts for in silico trials, offering a privacy-preserving path for virtual clinical research.^46^

### Variational autoencoder

**Figure 2:**
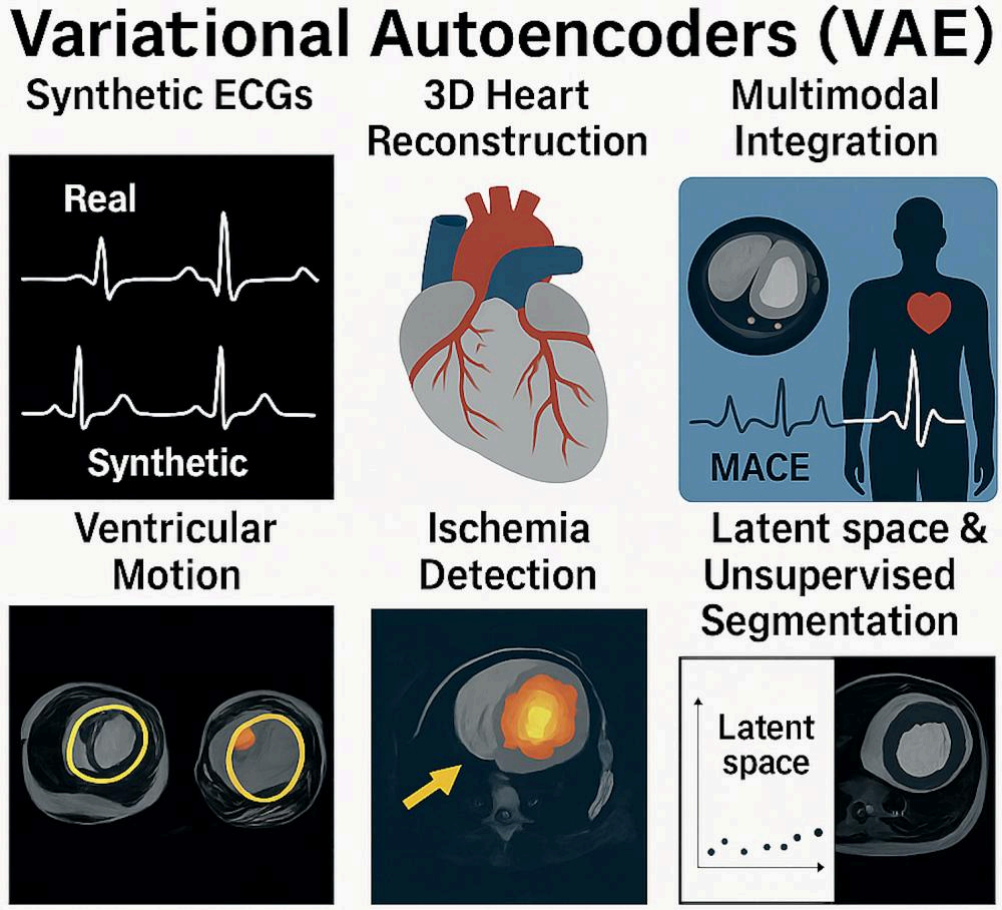
Applications of Variational Autoencoders in Cardiology. Applications of Variational Autoencoders in cardiology, including synthetic ECG generation, latent space encoding, multimodal integration (MRI and ECG), 3D heart reconstruction, ventricular motion modelling, ischaemia detection, and unsupervised segmentation. For architectural and functional details, see Supplementary Material (Sections  *S1*–S2). ECG, Electrocardiogram; VAE, Variational Autoencoder. All clinical imaging examples (echocardiography, MRI, CT, and X-ray) shown in this figure originate from anonymized real patient data obtained from the authors’ institutions and used under institutional permission.

Variational autoencoders (VAEs) have emerged as valuable tools in cardiovascular research due to their ability to generate synthetic data while maintaining interpretability. By learning compact internal representations-called latent spaces-VAEs can reconstruct realistic clinical signals and images from reduced data dimensions, helping to address issues of missing or noisy input. In electrocardiography, VAEs have been applied to synthesize realistic ECG waveforms, allowing for dataset expansion and more balanced model training, particularly in the context of arrhythmia classification tasks.^47^ This capacity to capture the underlying variability of cardiac signals also improves feature extraction, enhancing the interpretability of prediction models. In cardiac imaging, VAEs have been used to reconstruct three-dimensional heart structures from imaging datasets, facilitating identification of anatomical patterns linked to adverse outcomes, such as major adverse cardiac events (MACE).^48^ Furthermore, VAEs support multimodal integration-for example, combining cardiac MRI and ECG data to simulate synthetic patients with both anatomical and electrical coherence.^49^ Unlike black-box architectures, VAEs provide transparent latent encodings, which can be mapped back to clinical features-offering an added layer of trust for clinician end-users.^50^ Additional research is exploring their role in modelling ventricular motion, ischaemia detection on MRI, and hybrid unsupervised segmentation using VAE-GAN pipelines. Although still early in clinical deployment, VAEs represent a promising framework for scalable, interpretable, and multimodal data synthesis in cardiovascular medicine. *Figure 2* illustrates representative applications of VAEs across signals, anatomy, and unsupervised image analysis.

### Transformers

**Figure 3:**
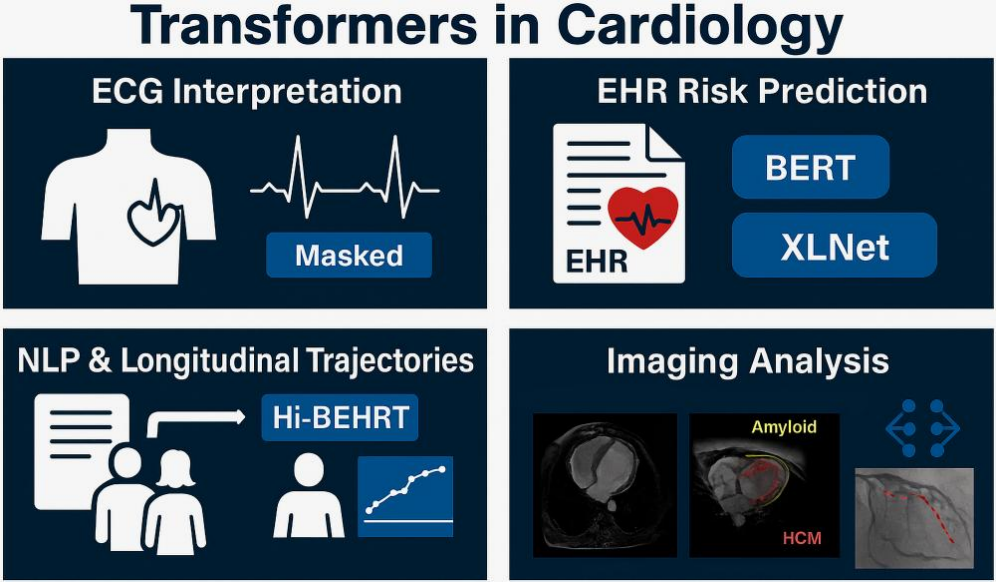
Applications of Transformer Architectures in Cardiology. Applications of Transformer models in cardiology, including ECG interpretation with masked attention, risk prediction from structured and unstructured EHR data, and longitudinal trajectory modelling using hierarchical architectures. For architectural and functional details, see Supplementary Material (Sections  *S1*–S2). BERT, Bidirectional Encoder Representations from Transformers; ECG, Electrocardiogram; EHR, Electronic Health Record; HCM, Hypertrophic Cardiomyopathy; Hi-BEHRT, Hierarchical BERT; XLNet, Generalized Autoregressive Pretraining. All clinical imaging examples (echocardiography, MRI, CT, and X-ray) shown in this figure originate from anonymized real patient data obtained from the authors’ institutions and used under institutional permission.

Originally developed for natural language processing, Transformer architectures have rapidly gained traction in cardiology due to their ability to model complex temporal relationships and handle diverse data types. Their core innovation-self-attention-allows the model to evaluate the relative importance of each input element, making them ideal for sequential and multimodal clinical tasks. In ECG analysis, Transformers have demonstrated excellent performance in classifying arrhythmias by treating waveforms as sequences of time-dependent events, much like words in a sentence. These models have achieved high accuracy in rhythm abnormality detection, especially when combined with convolutional layers that extract local features prior to sequence modelling.^51,52^ Recent lightweight versions, such as masked Transformers, improve computational efficiency while retaining diagnostic power.^53^ In electronic health record (EHR) modelling, Transformers have been used for advanced risk prediction and outcome stratification. Pretrained architectures such as BERT and XLNet, when fine-tuned on structured and unstructured cardiac EHR datasets, have outperformed conventional machine learning models in predicting 6-month mortality, identifying high-risk patients more accurately.^54,55^ These models have also extracted risk factors directly from free-text clinical notes with high precision,^56^ while hierarchical frameworks like Hi-BEHRT have captured long-range patient trajectories in longitudinal data.^57^ In the field of cardiac imaging, Transformers have improved cine MRI frame rates and resolution, enhancing motion analysis without compromising spatial quality.^58^ Vision Transformers (ViTs) have also been successfully trained to differentiate between cardiac amyloidosis and hypertrophic cardiomyopathy using MRI with higher specificity.^59^ In interventional cardiology, Transformer models have been employed to segment systolic wall abnormalities from cardiac catheterization X-rays, leveraging attention-based mechanisms to improve feature recognition.^60^ Together, these developments illustrate the adaptability of Transformer architectures in cardiovascular medicine. While most clinical applications are still emerging, their ability to integrate and interpret large volumes of sequential, structured, and unstructured data positions them as a promising foundation for next-generation decision support systems. *Figure 3* illustrates key areas of Transformer application across ECG interpretation, EHR modelling, and cardiovascular imaging.

Recent cardiovascular applications provide quantitative evidence of the performance gains associated with Transformer-based architectures. In a study using multimodal EHR data for 6-month mortality prediction, ClinicalBERT achieved an AUROC of 0.86, outperforming logistic regression (0.78) and random forests (0.81) on the same dataset. Similarly, for heart-failure hospitalization prediction, Transformers demonstrated a 12–14% increase in AUPRC compared with gradient-boosted trees and CNN baselines. In the ECG domain, the integration of synthetic AI-generated signals with Transformer classifiers improved arrhythmia detection accuracy by 5–8% and specificity by 6–10% relative to models trained without synthetic augmentation. These findings illustrate how pretrained language-model architectures, when adapted to structured, unstructured, and physiological cardiac data, can exceed the performance of traditional machine-learning methods in clinically relevant tasks.

### Autoregressive modelling

**Figure 4:**
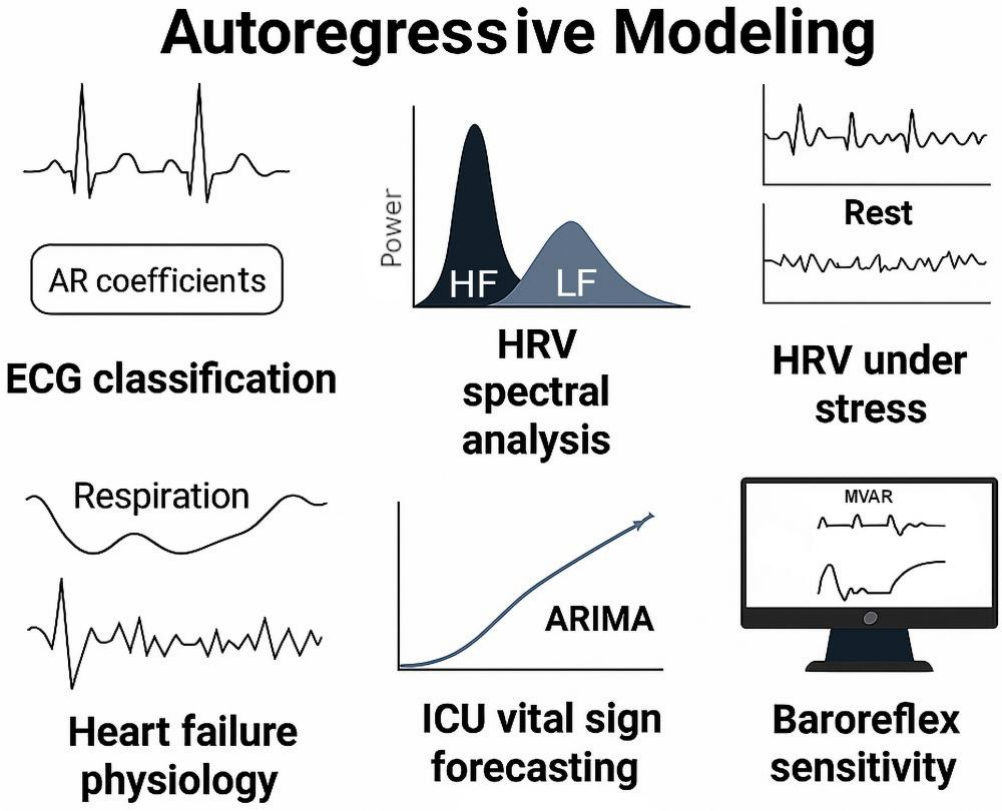
Autoregressive models in cardiology. Applications of autoregressive (AR) modelling in cardiology, including ECG-based rhythm classification, HRV spectral analysis, time-varying autonomic tracking, heart failure physiology, ICU vital sign forecasting, and baroreflex sensitivity estimation. For architectural and functional details, see Supplementary Material (Sections  *S1*–S2). AR, Autoregressive; ARIMA, Autoregressive Integrated Moving Average; ECG, Electrocardiogram; HRV, Heart Rate Variability; ICU, Intensive Care Unit. All clinical imaging examples (echocardiography, MRI, CT, and X-ray) shown in this figure originate from anonymized real patient data obtained from the authors’ institutions and used under institutional permission.

As illustrated in *Figure 4*, autoregressive (AR) models continue to play a foundational role in cardiovascular signal analysis, particularly where interpretability and physiologic transparency are essential. These models predict future values of a time-series based on past observations, enabling dynamic analysis of biologic rhythms and response patterns. In electrocardiography, AR models capture waveform dynamics, allowing for arrhythmia classification based on derived coefficients that distinguish normal and abnormal rhythm segments.^61^ Spectral analysis using AR frameworks is central to the assessment of heart rate variability (HRV), providing insights into autonomic nervous system function by quantifying low- and high-frequency components.^62^ Advanced variants, such as time-varying AR models, have been employed to track HRV responses under stress, capturing temporal patterns that differ from standard Fourier-based methods.^63^ AR modelling has also been applied in the context of heart failure, where it quantifies alterations in respiration and heart period variability associated with autonomic dysfunction.^63^ In intensive care, ARIMA (autoregressive integrated moving average) models have outperformed linear regression approaches in forecasting acute changes in vital signs following cardiac surgery, aiding early warning systems.^64^ Beyond single-signal analysis, multivariate autoregressive (MVAR) models support the joint assessment of interconnected physiologic variables. For example, bivariate MVAR modelling of blood pressure and heart rate enables estimation of baroreflex sensitivity, a key marker of cardiovascular regulation.^65^ These models often include feedforward and feedback components to reflect bidirectional influences between physiologic systems. Software platforms like CardioRVAR implement such frameworks for non-invasive evaluation of autonomic function. While modern deep learning models now often surpass AR methods in predictive accuracy for tasks like ECG interpretation and risk modelling,^66^ autoregressive approaches remain valuable due to their interpretability, physiologic grounding, and transparency. They continue to serve as reference models and baseline comparators in both research and clinical algorithm development.

### Diffusion models

Originally developed for generative tasks in computer vision, diffusion models are now being explored in cardiology for data synthesis, augmentation, and physiologic simulation. These models work by learning to reverse a noise-based degradation process, progressively reconstructing realistic clinical signals or images from random inputs.

In cardiac imaging, diffusion-based frameworks have been used to generate synthetic cardiac MRI datasets with high structural fidelity, enabling model training while protecting patient privacy.^67^ These synthetic images serve as data augmentation tools in low-sample or ethically sensitive contexts.

In electrocardiography, generative neural networks have been applied to synthesize ECG signals, enriching underrepresented arrhythmic patterns in benchmark datasets and supporting improved detection performance in rhythm classification task 150.^68^

Beyond signal generation, diffusion models have also been investigated for 3D reconstruction of cardiac activation maps from sparse 2D imaging slices.^69^ This approach supports analysis of mechanical activation delays, which are critical in heart failure and device planning. Early-stage research suggests diffusion-based generators may also simulate cardiac excitation wavefronts, offering a scalable, data-driven alternative to traditional physics-based electrophysiological simulations.^70^

Although most cardiology applications of diffusion models-remain at the research stage, their ability to synthesize realistic, privacy-compliant, and physiologically consistent data positions them as a complementary tool for simulation, diagnostic support, and model training.

**Figure 5:**
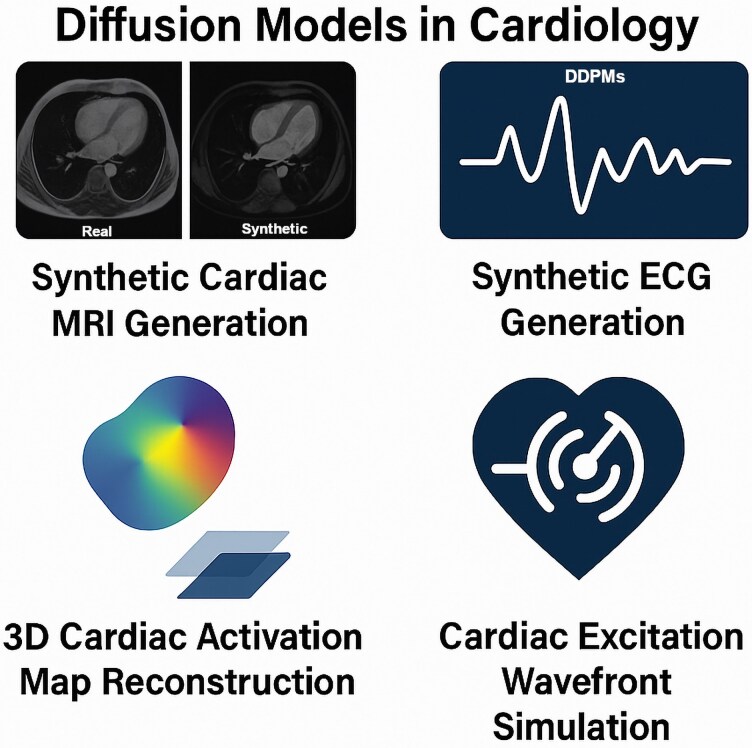
Diffusion models in cardiology. Applications of diffusion models in cardiology, including synthetic cardiac MRI and ECG generation, 3D reconstruction of activation maps, and simulation of cardiac excitation wavefronts. For architectural and functional details, see Supplementary Material (Sections  *S1*–S2). CRT, Cardiac Resynchronization Therapy; DDPM, Denoising Diffusion Probabilistic Model; ECG, Electrocardiogram; MRI, Magnetic Resonance Imaging. All clinical imaging examples (echocardiography, MRI, CT, and X-ray) shown in this figure originate from anonymized real patient data obtained from the authors’ institutions and used under institutional permission.

*Figure 5* illustrates key contributions of diffusion models to synthetic imaging, signal enrichment, and virtual physiologic reconstruction.

### Digital twins

Digital twins are emerging as transformative tools in cardiology, offering patient-specific, dynamic simulations of cardiac anatomy, physiology, and electrophysiology. These virtual replicas are built from multimodal clinical data-including imaging, electrocardiography, and laboratory and demographic inputs-and update in real time to mirror changes in patient condition.

In cardiac electrophysiology, digital twins have been applied to simulate arrhythmic behaviour and guide procedural planning for catheter ablation. Mechanistic models are now capable of incorporating real-time inputs and being calibrated to the specific electrical and structural features of individual patients.^71^ Broader applications are expanding into integrated AI and extended reality platforms, enabling digital twins to assist with procedural rehearsal, anatomical visualization, and risk projection across disciplines such as heart failure, congenital cardiology, and surgical planning.^72^

A notable example is the work from Johns Hopkins University, where digital twins have been developed to simulate therapy responses before treatment in complex arrhythmic cases.^73^ Although widespread clinical adoption remains limited, digital twin platforms are advancing rapidly and may become central to precision cardiovascular care, procedural training, and real-time decision support.

### Synthetic cohort simulators

In contrast to individualized digital twins, synthetic cohort simulators generate entire virtual populations that reproduce real-world clinical patterns. These simulators are used to model disease progression, simulate virtual clinical trials, and test interventions without involving real patient data. Synthetic cohorts are trained on real datasets and preserve statistical correlations between variables (e.g. age, risk factors, outcomes), while maintaining privacy by design. They enable robust in silico studies, particularly in scenarios where data access is limited or sensitive. For example, one application involved integrating data from multiple longitudinal studies to construct a synthetic cohort reflecting cardiovascular risk evolution across the lifespan. This model reproduced real-world trends and allowed for timeline-based risk analysis^74^. In another case, virtual patients derived from risk calculators were used to test the alignment between predicted and observed outcomes, offering a means to refine predictive models with embedded uncertainty.^74^The MedalCare-XL project produced over 16 000 synthetic 12-lead ECGs, representing a spectrum of normal and pathological cases. This dataset supports benchmarking of machine learning models without reliance on identifiable clinical recordings.^75^ Other notable work includes the generation of detailed synthetic thoracic aorta models for use in device testing and preclinical simulations.^76^ Together, digital twins and synthetic cohorts offer complementary tools-individual-level precision vs. population-scale modelling.

**Figure 6:**
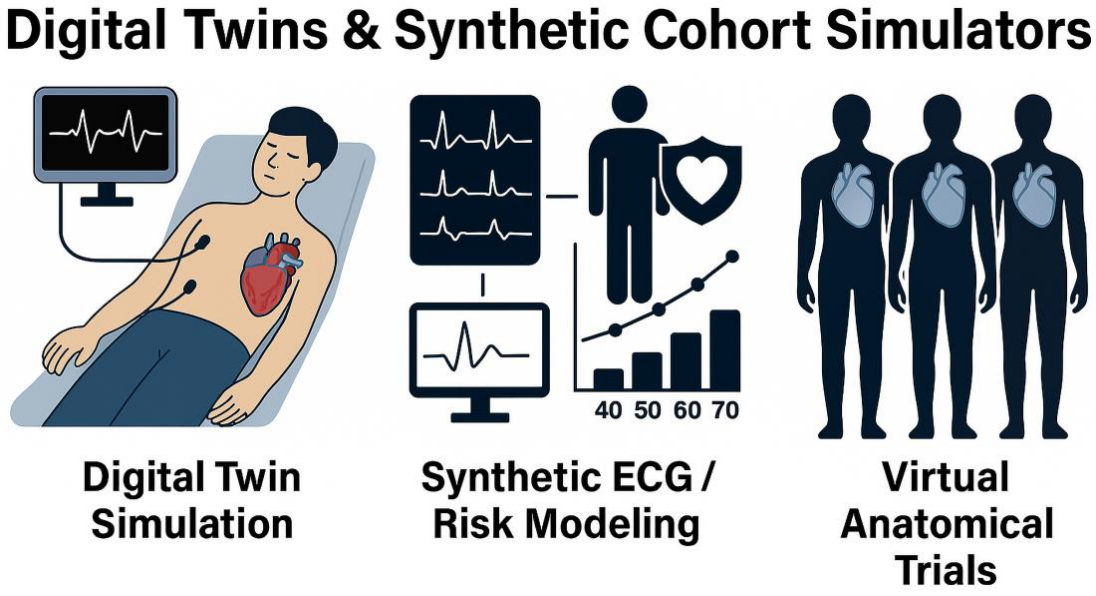
Digital twins and synthetic cohort simulators. Digital twins and synthetic cohort simulators in cardiology. Panels illustrate patient-specific simulation for procedural planning, synthetic ECG generation for risk modelling, and population-scale virtual anatomical trials. For architectural and functional details, see Supplementary Material (Sections  *S1*–S2). ECG, Electrocardiogram. All clinical imaging examples (echocardiography, MRI, CT, and X-ray) shown in this figure originate from anonymized real patient data obtained from the authors’ institutions and used under institutional permission.

*Figure 6* highlights this distinction and illustrates how both approaches support different tiers of data-driven cardiology.

## Challenges and opportunities

### Challenges

Despite their promise, synthetic AI methods face distinct limitations that currently constrain clinical application.

*Generative Adversarial Networks* (*GANs*) generate realistic cardiac images and signals but are affected by training instability, mode collapse, and artefact generation.^10,77^ Clinical validation is limited, and their adversarial nature complicates interpretability and regulatory acceptance.

*Variational Autoencoders* (*VAEs*) enhance data representation and augmentation but often yield blurred outputs and lower fidelity than GANs.^78,79^ While they improve model transparency and capture ECG morphology variation, clinical validation remains incomplete.

*Transformers* have advanced ECG and EHR modelling but demand large, high-quality datasets and are computationally intensive.^53^ Performance may drop with sparse or heterogeneous inputs.^80–82^ Despite accuracy in arrhythmia detection, interpretability and infrastructure requirements limit widespread use.^83^

*Autoregressive models*-widely used in time-series analysis-are constrained by linear assumptions and noise sensitivity. These issues, recognized in ECG modelling for decades,^84^ limit performance in complex tasks like wearable-based arrhythmia detection, where deep learning models better capture non-linear dynamics.^85,86^ While valued for transparency and physiologic plausibility, their role is narrowing as data complexity increases.

*Diffusion models,* though promising, remain experimental in cardiology. Their training and inference are computationally demanding.^87^ They require large datasets^88^ and may overfit, raising concerns about privacy and bias^89^-hindering scalability and integration into routine workflows.

*Digital Twins* offer individualized modelling but face translational barriers. Most remain static or cohort-based, lacking dynamic updating.^90^ Multiorgan twins remain aspirational due to complexity. Even advanced models for surgical planning often require manual correction.^91^ Decision-making still depends on clinician oversight, and models like CircAdapt show predictive potential but limited precision.^92^ Broad adoption requires improved fidelity, interoperability, and validation.

*Synthetic cohort simulators* also pose challenges. While plausible, their signals often fail to fully reflect clinical complexity.^93^ Models trained solely on synthetic data may lack generalizability. Simulators may reproduce biases or oversimplify profiles,^94^ and may underrepresent minority groups.^95^ Rare events remain especially hard to model accurately.

Finally, the absence of standardized evaluation frameworks, transparent labelling, and auditing mechanisms creates risk. Even high-fidelity synthetic datasets must be assessed for clinical relevance. Without traceability, flawed data could compromise downstream prediction models, trial simulations, or clinical decisions.^96^

In sum, while synthetic AI offers major opportunities for cardiovascular innovation, each approach brings distinct and shared limitations. Addressing these will require continued technical advancement, validation, ethical oversight, and regulatory evolution.

#### Lack of provenance and auditability

An additional challenge relates to the lack of provenance of synthetic data outputs. Unlike conventional clinical datasets, synthetic data often lack a transparent audit trail, making it difficult to trace how specific samples were generated or which training inputs influenced a given output. This absence of data lineage complicates model validation, reduces interpretability, and limits the ability to reconstruct the generation process in case of errors or safety concerns. Moreover, because synthetic generators may incorporate patterns learned from multiple patients without retaining identifiable information, the chain of custody for upstream data sources becomes fragmented or undefined. These provenance gaps have important implications for regulatory approval, reproducibility, and clinical governance, underscoring the need for standardized metadata, generation logs, and traceability protocols in future synthetic AI systems.

### Ethical opportunities and ethical challenges in synthetic AI

The integration of synthetic AI into cardiovascular medicine introduces not only technical advancements but also important ethical considerations. On the opportunity side, synthetic data generation offers notable privacy benefits. By creating patient-like datasets without using real identities, synthetic AI can lower re-identification risks and facilitate secure data sharing without violating confidentiality.^97^

In silico trials based on synthetic cohorts may reduce ethical burdens related to patient recruitment, especially among vulnerable populations.^98,99^ Democratizing access to synthetic data could also reduce research disparities by enabling participation from smaller centres and resource-limited settings.^100,101^ Combining synthetic data with federated learning approaches-such as FedSyn, which trains models across decentralized datasets without sharing raw data-may enhance privacy, streamline regulation, and promote collaboration.^102–105^

However, these advantages come with ethical challenges. Poorly validated synthetic data may perpetuate biases from source datasets or introduce artefacts that distort clinical reasoning.^106^ Transparency around provenance, quality, and intended use is essential to maintaining clinician and public trust.^107^ Questions regarding authorship, ownership, and accountability must be resolved, particularly as synthetic models begin to influence medical decision-making.

Regulatory frameworks have yet to fully address the ethical risks posed by synthetic AI. These include data misuse, algorithmic discrimination, and reduced accountability when harm results from machine-generated outputs.^108^

A responsible path forward must include not only technical validation but also robust ethical oversight. Key elements include clear labelling of synthetic vs. real data, explicit consent protocols where applicable, and engagement of ethicists, clinicians, and patients. Ensuring that innovation proceeds alongside accountability will be critical to preserving ethical standards in cardiovascular research and care.

## Future perspectives

As synthetic AI matures, its potential to reshape cardiology becomes increasingly clear. Future applications of GANs may extend beyond image and ECG augmentation to simulating entire disease trajectories, supporting real-time decisions, and enabling personalized trial designs.^38,109^ Achieving clinical deployment will require enhanced training stability, conditional frameworks, and robust validation protocols. VAEs are expected to contribute to large-scale phenotype modelling, generation of virtual anatomical or physiological cohorts, and unsupervised discovery of latent disease states.^48,49^ Hybrid VAE architectures may further improve fidelity, interpretability, and multimodal integration across imaging, signal, and omics domains. Transformers, with their strength in modelling long-range dependencies, are poised to advance ECG analysis, EHR interpretation, and imaging workflows.^82,110,111^ Future directions include cardiovascular-specific pretraining, real-time application variants, and improved interpretability tools such as attention heatmaps.^53^

Autoregressive models, while more established, may gain renewed relevance through hybrid approaches. Their transparency and utility in structured physiological forecasting support their use in early warning and ICU monitoring.^112^ Diffusion models, though early-stage, hold strong promise for high-resolution synthesis of cardiac images, ECGs, and virtual cohorts.^69,113^ Advancements in inference speed, fidelity, and validation could enable disease-specific generation, cardiac activation simulation, and privacy-preserving data sharing. Digital twins represent a particularly ambitious horizon. Future models will integrate imaging, biosensors, EHR, and genomic data into adaptive, continuously updating patient-specific simulations.^90–92^ Success will depend on progress in multiscale modelling, real-time calibration, and regulatory alignment.

Synthetic cohort simulators are expected to become standard tools for epidemiological modelling, in silico trial design, and device evaluation. Increasing realism-especially among underrepresented populations-and standardized metrics will be critical for regulatory and policy integration.^114,115^

In summary, synthetic AI in cardiology is moving from experimental to translational phases. Realizing its full potential will demand technical innovation, external validation, ethical oversight, and structured regulation. If achieved, it may offer scalable, individualized, and ethically sound solutions to longstanding challenges in cardiovascular care and research.

## A glance at the market

While AI-based cardiovascular tools such as arrhythmia detection systems and imaging triage platforms^116–121^ have entered clinical use, synthetic AI technologies-those that generate synthetic patients, signals, or anatomical models-remain largely experimental. As of this writing, no synthetic AI platform has achieved independent CE-marking or FDA clearance for direct diagnostic or therapeutic use in cardiovascular care.

In the domain of synthetic ECG generation, academic initiatives have yielded high-quality datasets. For example, MedalCare-XL has released a database of over 16 900 synthetic 12-lead ECGs generated via electrophysiological simulations.^93^ Similarly, the DeepFake ECG project has demonstrated privacy-preserving ECG synthesis using GANs.^122^ However, both platforms remain research-oriented and have not received regulatory certification.

In synthetic cardiac imaging, platforms like SynthRAD and MEDDiff have explored the use of GANs and diffusion models to generate synthetic cardiac MRI and CT datasets.^123^ These tools aim to support AI training and validation but are not approved for diagnostic application.

Digital twin technologies are marginally more advanced, although they too remain under clinical evaluation. Siemens Healthineers has developed a digital twin cardiology platform that is undergoing testing.^124^ Meanwhile, Dassault Systèmes’ Living Heart Project seeks to simulate patient-specific cardiac structure and function.^125^ Neither has received regulatory clearance for routine clinical use.

In the synthetic cohort space, the open-source platform Synthea (The MITRE Corporation) provides virtual patient datasets for research, training, and algorithm validation.^126^ Nonetheless, synthetic cohorts are not currently accepted as real-world evidence for regulatory submissions.

Overall, while synthetic AI holds considerable potential for research and education, clinical adoption will depend on substantial progress in validation, regulatory engagement, and workflow integration.

## Regulatory frameworks, guidelines, and workflow integration

### Regulatory considerations and path to clinical validation

The clinical deployment of synthetic AI will require a well-defined regulatory framework that ensures safety, reliability, and ethical compliance.

#### European union

The European Union's Artificial Intelligence Act (AI Act),^127,128^ effective as of 1 August 2024, classifies AI systems by risk level. Healthcare applications are designated ‘high-risk’ and subject to mandatory conformity assessments, transparency requirements, and human oversight. Compliance obligations will apply 24 to 36 months after enactment, depending on system type.

#### United States

The U.S. Food and Drug Administration (FDA) has explored regulatory flexibility through the Software Precertification Pilot Program.^129^ Though concluded in 2022, the program offered insights into managing adaptive software-based medical tools, including AI. The FDA has also published position papers proposing a total product lifecycle model for the oversight of AI/ML-based software,^130^ highlighting the need for new regulatory paradigms as technologies evolve.

### Integration into clinical guidelines and workflow

#### Professional societies

At present, no major cardiology society has issued formal guidelines addressing synthetic AI. However, leading organizations are actively working toward structured integration of AI systems.

The European Society of Cardiology (ESC) has launched the AI Hub,^131^ aiming to develop tools for clinical prediction, risk modelling, and data standardization. In 2024, the ESC hosted a Cardiovascular Round Table focused exclusively on AI, working toward a European roadmap for safe and effective deployment.^132^

In the United States, the ACC and AHA have jointly endorsed AI’s role in improving cardiovascular outcomes. heir scientific statement highlights diagnostic and risk prediction applications, aligning with key points by Lüscher *et al*.^133^ in EHJ, which emphasize structured and unstructured data integration, continuous validation, and ethical implementation.^133–135^ At the recent ACC. 2025 Scientific Session, new clinical applications of AI were presented, further highlighting its expanding role in cardiovascular care.^136^

#### Clinical workflow integration

Integrating synthetic AI into clinical workflows goes beyond technical deployment. As Ahmed *et al*.^137^ note, redesigning care pathways is key to ensuring usability and interoperability. Chen *et al*.^138^ show how AI aids real-time decisions, while Patel *et al*.^139^ stress validation and regulation. Though focused on AI broadly, these principles apply to synthetic AI, which presents unique challenges. As highlighted by Skandarani *et al*.^77^ generative approaches like GANs offer promise in cardiology but require tailored evaluation and cautious implementation.

#### The role of clinical oversight in synthetic data generation

Integrating synthetic AI into clinical workflows extends beyond deployment. Alignment with clinical priorities, streamlined usability, and thoughtful design have been identified as critical success factors for effective implementation.^77^ Broader literature supports AI’s contribution to outcome improvement and system-wide integration across cardiovascular care.^140^ However, synthetic AI introduces specific challenges. As shown in recent evaluations, synthetic datasets require rigorous validation to ensure safety, clinical relevance, and trustworthiness in decision-making environments.^34^

#### Team-implementation multidisciplinary approach

The Team-Implementation Multidisciplinary Approach (TIMA)^19^ recently proposed in the context of synthetic data generation using CTGANs, offers a structured framework for integrating clinician expertise into model validation. In this model, physicians not directly involved in model development are tasked with evaluating the realism, logical consistency, and population-level representativeness of synthetic datasets. Their iterative contributions resulted in synthetic outputs with high scores for coverage, novelty, and coherence-benchmarks unlikely to have been met without embedded clinical oversight. Going forward, structured involvement of clinicians in synthetic AI workflows should be formalized in both regulatory frameworks and institutional governance. In cardiovascular applications, where synthetic data must replicate complex physiological patterns, this type of oversight is critical to ensure both safety and trust.

## Conclusions

This review was not written to close a discussion but to open one-to spark curiosity in all cardiologists, from academic researchers to front-line clinicians. For the former, it offers technical synthesis; for the latter, a bridge to engagement. Synthetic AI is not an abstraction-it is a practical opportunity for workflow support, diagnostic augmentation, and clinical insight.

If conventional AI resembles a self-driving car, synthetic AI is a high-performance machine: precise, powerful, but dependent on skilled hands. It won’t replace cardiologists-it will extend them.

We acknowledge the limitations. But progress is accelerating. As engagement grows, so will impact methods will mature. Realism and clinical alignment will improve.

Synthetic AI should not be feared. It should be understood, tested, and-where appropriate-embraced. It is a tool, not a threat. And cardiologists are not passengers in this transformation-they are drivers. This review invites them to take the wheel.

## Supplementary Material

oeag026_Supplementary_Data

## Data Availability

No datasets were generated or analysed during the current study. This manuscript is a narrative review and all data discussed derive from previously published studies, which are appropriately cited within the article.

## Author note on use of grey literature

This review includes a limited number of references from company websites and commercial sources^114,117–121,124–126,131,132,134–136^ to describe the current landscape of available synthetic AI tools. As no peer-reviewed evaluations exist for many early-stage products, these references were included exclusively in the market overview section to inform readers of emerging technologies.
