# Supplementary material for: Synthetic artificial intelligence in cardiology: from generative models to clinical applications
Source: Eur Heart J Open. 2026 Mar 1;6(2):oeag026. doi: 10.1093/ehjopen/oeag026 (PMC13070426; doi:10.1093/ehjopen/oeag026)
Supplement: oeag026_Supplementary_Data [file oeag026_supplementary_data.zip › Supplementary Material R1 tracked . docx.docx]

**Supplementary Material.**

### **Section 1 – Graphical Abstract Legend: Synthetic AI in Cardiology**

This figure summarizes the central theme of the manuscript: **synthetic artificial intelligence (AI)** as a high-performance clinical tool that enhances,rather than replaces,cardiologist expertise. The image of a cardiologist driving a high-performance vehicle symbolizes the role of human oversight and clinical judgment in steering AI deployment.

Key components of synthetic AI are visually represented: a **researcher in a lab** interacts with generative tools, emphasizing the translation of innovation from bench to bedside; a **stylized AI brain** highlights the central role of synthetic intelligence in transforming complex medical data into actionable outputs. **Silhouettes of virtual patients and synthetic cohorts** illustrate the bridging of research and care, while the figure overall introduces core synthetic AI architectures, including **GANs, VAEs, and diffusion models**, as integral components of next-generation cardiovascular medicine

**Section 2: Technical Foundations of Synthetic AI Methods**

This section expands on the model architectures referenced in the main manuscript, providing readers with an overview of how these generative models operate, train, and apply to cardiovascular data.

**Supplementary Figure S1 – Generative Adversarial Network (GANs)Technical Foundations.**

This figure presents the fundamental structure of a Generative Adversarial Network (GAN), a model used to produce synthetic data that mimics the statistical properties of real datasets. The figure is organized into three main sections:

On the left, the Generator receives a random input (often noise or a latent vector) and transforms it into a synthetic output-such as an ECG waveform, a cardiac MRI image, or a tabular patient profile.

In the center, the Discriminator evaluates both real examples from the training data and the synthetic outputs. It assigns a probability to indicate whether each input is real or generated.

On the right, clinical examples show synthetic outputs used in cardiology

The GAN is trained through an adversarial process: the Generator improves by learning to fool the Discriminator, while the Discriminator learns to catch increasingly realistic fakes. This loop continues until the synthetic outputs become nearly indistinguishable from the original data.

Conceptually, GANs are powerful because they do not require explicit labels and can generalize distributions from limited samples. Clinically, they are used to generate data when real examples are rare, imbalanced, or ethically sensitive.

**Supplementary Figure S2 – Variational Autoencoders (VAEs)Technical Foundations.**

This figure illustrates the structure and application of Variational Autoencoders (VAEs), a model used to compress and reconstruct data while learning a structured latent representation. The diagram includes:

On the left, an Encoder processes clinical inputs-ECG waveforms, MRI slices, or tabular data-and compresses them into a latent representation (a compact vector).

In the center, this latent space is depicted as a soft cloud or distribution. Each point represents a potential encoding, capturing variations in physiology such as morphology, rhythm, or disease burden.

On the right, a Decoder reconstructs the input from the latent code, producing a realistic ECG, anatomical model, or multimodal signal.

Unlike traditional autoencoders, VAEs encode inputs into distributions rather than fixed points, enabling controlled sampling and generation. This structure facilitates the generation of new data that is both diverse and physiologically plausible.

Clinically, VAEs are useful for expanding training datasets, modeling 3D cardiac anatomy, and linking latent features to disease traits. They can also fuse different modalities (e.g., ECG + MRI) into unified synthetic outputs, supporting hybrid modeling. Their interpretability and generative capacity make them ideal for both exploratory research and dataset augmentation in AI pipelines.

**Supplementary Figure S3 –Transformers Technical Foundations.**

This figure explains the architecture of Transformer models, designed for processing sequential and heterogeneous data. The diagram shows:

On the left, clinical inputs such as ECG waveforms, patient timelines, or text notes are broken into tokens, each representing a discrete element.

In the center, these tokens enter the self-attention mechanism of the Transformer. Here, the model learns which tokens are most relevant to each other. Tokens "attend" to all others, creating weighted relationships that guide downstream predictions.

On the right, the processed sequence feeds into a prediction head-used for arrhythmia classification, outcome prediction, or imaging enhancement. Attention maps enable interpretability by highlighting which features were most influential.

Transformers are distinguished by their ability to process entire sequences in parallel and model long-range dependencies. This makes them suitable for complex tasks involving time-series ECG data, EHR-based prognosis, and multimodal integration.

**Supplementary Figure S4 – Autoregressive (AR) and Multivariate Autoregressive (MVAR) Models Technical Foundations.**

This figure describes Autoregressive (AR) and Multivariate Autoregressive (MVAR) models used in cardiovascular signal analysis. The layout includes:

On the left, a single time-series input (e.g., ECG, HRV, or BP) where the model predicts the next value based on prior observations (e.g., t–1, t–2, t–3).

In the center, a forecasted signal is shown. AR models estimate short-term physiologic trends-used for ICU monitoring, HRV prediction, and early warning systems.

On the right, MVAR logic is depicted using coupled signals (e.g., HR ↔ BP), where the model captures bidirectional influences, such as baroreflex dynamics.

Also included are examples of outputs: HRV power spectra, baroreflex sensitivity (BRS) metrics, and time-varying AR graphs that update in real time. These models are computationally efficient and interpretable, making them ideal for embedded systems and physiologic assessment.

Clinically, AR models support real-time monitoring, transparent prediction, and physiologic interpretation. They are often used as benchmarks or embedded components in more complex AI pipelines, particularly where physiological validity is essential.

**Supplementary Figure S5 –Diffusion Models Technical Foundations.**

This figure illustrates the generative process behind diffusion models, which synthesize data by reversing a stepwise noise process. The visual flow includes:

On the top left, training inputs (e.g., ECGs, cardiac MRI) are progressively noised across multiple steps, producing degraded outputs.

In the center, a fully noised signal represents the model's starting point for generation.

On the right, the model applies a reverse denoising process, gradually reconstructing a realistic output-such as a synthetic ECG, MRI segment, or anatomical model.

Unlike GANs, diffusion models do not rely on adversarial training but instead use probabilistic estimation of data transitions. Their ability to preserve spatial and temporal structure makes them highly effective for high-fidelity signal generation.

Diffusion models are well-suited to contexts where output realism, data variety, and patient privacy are critical.

**Supplementary Figure S6 –Digital Twins Technical Foundations** .

This figure presents the architecture of digital twin frameworks in cardiology. These models simulate individual patients using multimodal clinical data to support personalized decision-making.

On the left, patient-specific data-including ECGs, cardiac imaging, and lab values-are collected.

In the center, these inputs feed a simulation engine, configured to model that patient's electrophysiology, mechanics, and disease progression.

On the right, digital twin outputs support ablation planning, therapy personalization, and risk simulation.

Digital twins are dynamically updated and physiologically grounded.Their value lies in offering a predictive, non-invasive platform for individualized care.

**Supplementary Figure S7 –Synthetic Cohorts Technical Foundations** This figure describes synthetic cohort simulators, which generate artificial populations statistically matched to real-world data. The layout includes:

On the left, data sources such as Electronic Health Records (EHRs), population registries, or risk calculators provide reference distributions.

In the center, a simulation engine synthesizes new patient records without direct individual linkage.

On the right, use cases are shown: ECG libraries (e.g., MedalCare-XL), aortic models, and in silico trial populations.

These systems enable privacy-preserving simulation, scalability, and validation of algorithms in diverse populations. Synthetic cohorts are especially useful for rare disease modeling, digital trials, and regulatory-grade data augmentation.

**Section 3. Search Strategy**

A structured search strategy was used to identify publications related to synthetic and generative artificial intelligence in cardiovascular medicine. The search adhered to PRISMA principles and included the following components:

### **Databases**

- **PubMed/MEDLINE**
- **Scopus**

### ***Search Terms***

A combination of MeSH terms and free-text keywords was used. Boolean operators AND/OR combined the concepts of synthetic AI and cardiology. The primary search terms included:

- “synthetic data”
- “synthetic artificial intelligence”
- “generative AI”
- “generative models”
- “GAN” OR “Generative Adversarial Network”
- “VAE” OR “Variational Autoencoder”
- “diffusion model”
- “autoregressive model”
- “digital twin”
- “synthetic cohort”
- “cardiology”
- “cardiovascular”
- “ECG synthesis”
- “medical image synthesis”

These terms were combined as follows:
**(synthetic OR generative OR “digital twin” OR GAN OR VAE OR diffusion OR autoregressive) AND (cardiology OR cardiovascular OR ECG OR “cardiac imaging”)**

### ***Time Range***

- The search covered the period **January 2014 – April 2025**, reflecting the era in which modern generative models emerged.

### ***Inclusion Criteria***

- Studies applying synthetic or generative AI to cardiovascular medicine.
- Preclinical or clinical research involving synthetic imaging, ECGs, physiological simulations, or synthetic cohorts.
- Original articles, technical papers, methodological papers, and systematic reviews.
- Articles in English.

### ***Exclusion Criteria***

- Studies outside the cardiovascular field.
- Articles using only conventional (non-generative) AI without a synthetic component.
- Editorials, commentaries, short news items without primary data.
- Duplicate records.

### ***Screening Process***

Two authors independently screened titles and abstracts. Full texts were reviewed when eligibility was unclear. Disagreements were resolved by consensus.

**Section 4. Existing Reviews on Synthetic and Generative AI in Healthcare and Cardiology**

# **Table 4.1. Reviews on Synthetic Data and Generative AI in Healthcare (2024–present)**

| **First author** | **Year** | **Title** | **Journal / Source** | **Type of review** | **Main focus / Scope** |
| --- | --- | --- | --- | --- | --- |
| Pezoulas VC | 2024 | Synthetic data generation methods in healthcare: a review on open-source tools and methods | Computational and Structural Biotechnology Journal | Narrative / methods-focused review | Overview of synthetic data generation methods and open-source tools across healthcare data modalities. |
| Rujas M | 2024 | Synthetic data generation in healthcare: a scoping review | Computer Methods and Programs in Biomedicine | Scoping review | Healthcare domains where synthetic data are generated, motivations, future uses, limitations, and data types. |
| Rujas M | 2024 | Synthetic Data Generation in Healthcare: A Scoping Review of Reviews on Domains, Motivations, and Future Applications | medRxiv preprint | Scoping review of reviews | Synthesis of existing reviews on synthetic data in healthcare, covering domains, motivations, and applications. |
| Akpinar MH | 2024 | Generative adversarial networks in healthcare: a systematic review of image- and signal-based studies | Journal of Imaging Informatics in Medicine / similar (systematic review) | Systematic review | Applications of GANs for medical image and signal generation across clinical domains. |
| Shanmugam D | 2024 | Generative Artificial Intelligence in Medicine | Annual Review of Biomedical Data Science | Narrative review | Comprehensive overview of generative AI models and use cases in medicine. |
| Rouzrokh P | 2025 | A Current Review of Generative AI in Medicine | Journal of Medical Internet Research (or similar) | Narrative review | Balanced overview of generative AI applications, benefits, and challenges in clinical medicine. |
| Zhang P | 2024 | Generative AI in Medicine and Healthcare: Moving Beyond Hype | Future Internet | Narrative review | Integration of generative AI into healthcare workflows, opportunities and risks. |
| Loni M | 2025 | A review on generative AI models for synthetic medical text, time series, and longitudinal data | npj Digital Medicine | Scoping review | Models for generating synthetic health records (text, time series, longitudinal data) and their evaluation. |
| Foraker R | 2025 | Understanding synthetic data: artificial datasets for real-world healthcare | BMJ Evidence-Based Medicine | Narrative / conceptual review | Conceptual framework on statistical validity, privacy, and use of synthetic data in healthcare delivery. |
| van Dijk B | 2024 | A Novel Taxonomy for Navigating and Classifying Synthetic Data in Healthcare | Journal / conference paper (taxonomy study) | Conceptual review / taxonomy | Taxonomy of synthetic data in healthcare by data proportion, data modality, and data transformation. |

# **Table 4.2. Cardiology-Focused Reviews on Synthetic / Generative AI (2022–2025)**

| **First author** | **Year** | **Title / Scope** | **Limitations of the review** | **What our Review adds** |
| --- | --- | --- | --- | --- |
| Skandarani | 2022 | Generative Adversarial Networks in Cardiology – overview of GAN applications in CV imaging and signals | Focuses exclusively on GANs; no coverage of VAEs, diffusion models, transformers, digital twins, or synthetic cohorts | Broader synthesis: includes all generative families and simulation-based synthetic AI relevant to cardiology |
| Thangaraj | 2024 | Cardiovascular care with digital twin technology in the era of generative AI – review of cardiac digital twins | Covers only digital twins; no discussion of generative models, data synthesis, ECG/image generation, or synthetic cohorts | Integrates digital twins within a unified synthetic AI framework together with generative and probabilistic models |
| Hanycz | 2025 | Generative AI for cardiac ECG interpretation – practical review | Restricts analysis to ECG generative models; no imaging, no structural models, no synthetic populations | Places ECG generation within the wider synthetic AI landscape across imaging, risk prediction, simulation, and cohorts |
| Zanchi | 2025 | Synthetic ECG signals generation – scoping review | Method-specific, ECG-only; does not address cardiology imaging, multimodal synthesis, or digital twins | Provides a cross-modality perspective including ECG, imaging, anatomical modeling, and virtual populations |

**Section 5:** **In-Depth Figure Interpretations from the Main Text: AI and Cardiology Use Cases.**

***Supplementary Legend to Figure 1****-* ***Applications of Generative Adversarial Networks in Cardiology.***

This legend expands on **Figure 1** in the main manuscript, presenting twelve key applications of Generative Adversarial Networks (GANs) in cardiology. These panels illustrate how GANs support image generation, segmentation, signal synthesis, artifact correction, and cohort simulation. Each panel represents an active or emerging use case across imaging, electrophysiology, risk prediction, and virtual research.

**First Row Left Panel**: This is a real LGE cardiac MRI image showing a short-axis view of the left ventricle without visible scar tissue. It serves as the input image for ScarGAN, which is trained to generate synthetic myocardial scars for use in data augmentation and training of segmentation algorithms. The panel illustrates the baseline anatomy prior to any GAN-based modification.

**First Row Middle Panel**: This image shows a chest X-ray overlaid with segmentation masks delineating anatomical structures such as the heart and lungs. The panel illustrates the use of GANs in medical image segmentation, where generative models can either produce synthetic labeled data or refine segmentation boundaries. This application is particularly valuable in tasks requiring large-scale annotated chest X-ray datasets, such as cardiothoracic disease screening.

**First Row Right Panel**: This panel presents a schematic representation of an electrocardiogram (ECG), included to illustrate the potential application of Generative Adversarial Networks (GANs) in ECG signal modeling. It symbolically highlights how GANs can be trained on physiological signals to synthesize realistic cardiac traces. These synthetic signals are used to augment datasets, improve algorithm performance for arrhythmia classification, and support privacy-preserving data generation.

**Second Row Left Panel**: This panel shows a Transesophageal Echocardiography (TEE) image with overlaid segmentation masks, illustrating the use of GANs in echocardiographic image analysis. GANs can support TEE segmentation either by generating synthetic labeled datasets or by refining segmentation outputs through adversarial learning. Given the procedural importance of TEE in structural heart disease and device placement, GAN-based segmentation offers promising tools for automation and decision support in interventional cardiology.

**Second Row Center Panel**: This panel shows an image of the coronary arteries, likely derived from coronary angiography or CT coronary angiography (CTCA), with highlighted vessel segments. It illustrates how GANs can support coronary analysis through synthetic data generation, enhanced vessel segmentation, and modality translation. These applications enable improved detection of stenosis, plaque characterization, and potentially even functional assessment (e.g., FFR prediction), enhancing diagnostic accuracy and planning in coronary artery disease.

**Second Row Right Panel**: This panel shows a cardiac MRI with overlaid segmentation of myocardial scar tissue. Unlike ScarGAN, which synthesizes scarred images for data augmentation, this panel illustrates a GAN-based segmentation approach used to delineate scar regions from real late gadolinium enhancement (LGE) MRI scans. GANs here serve to enhance the precision and morphological accuracy of scar detection, aiding in infarct quantification and clinical decision-making.

**Third Row Left Panel**: This panel presents a myocardial strain map, representing regional cardiac deformation such as longitudinal or circumferential strain. GANs are applied here to enhance or synthesize physiologically accurate strain patterns, supporting tasks like data augmentation, noise reduction, and resolution enhancement. This application reflects the extension of GAN-based modeling from anatomy to cardiac function, enabling improved characterization of subtle myocardial dysfunction in clinical and research settings.

**Third Row Center Panel**: This panel illustrates the use of GANs for clinical risk modeling and therapy guidance in cardiology. GANs contribute by generating synthetic patient data for training robust risk models, simulating treatment outcomes, and supporting decision-making through patient-specific trajectory modeling. These approaches enable more accurate stratification and therapy optimization, particularly in scenarios with limited labeled data or rare adverse outcomes. This represents a shift from anatomical synthesis to clinical translation, aligning generative AI with personalized cardiovascular care.

**Third Row Right Panel**: The panel illustrates a cardiac image improved through GAN-based denoising and motion correction. GANs are trained to restore degraded or noisy images by suppressing artifacts while preserving anatomical accuracy. This is particularly important in modalities like MRI, CT, and echocardiography, where motion from cardiac cycles and respiration can obscure critical structures. GAN-based restoration enhances image interpretability and improves the reliability of downstream analyses such as segmentation and quantification.

**Fourth Row, Left Panel**: This panel illustrates calcium deblooming in CT coronary angiography using GANs. Calcium blooming artifacts can exaggerate the appearance of coronary calcifications, obscuring the vessel lumen and impeding accurate stenosis assessment. GANs are trained to suppress these artifacts and reconstruct more anatomically faithful images, thereby improving the diagnostic value of CTCA. This application demonstrates how generative models can enhance image quality and enable more precise evaluation of coronary artery disease.

**Fourth Row, Middle Panel**: This panel illustrates the generation of a synthetic patient cohort using GANs. By learning the complex distribution of real-world cardiovascular data, GANs can create realistic yet anonymized patient instances, including imaging, physiological signals, and clinical features. These synthetic cohorts can be used for AI model training, validation, and regulatory submission while preserving patient privacy. This approach enables robust data augmentation, addresses population imbalance, and supports scalable, reproducible research in digital cardiology.

**Fourth Row, Right Panel**: This panel represents the use of GANs in virtual clinical trials. By generating realistic synthetic patient cohorts and modeling treatment effects over time, GANs enable the simulation of entire clinical studies. These virtual trials can be used to evaluate therapies, predict outcomes, and support regulatory decision-making , all while reducing the costs and ethical constraints of traditional trials. GANs facilitate scenario testing, rare disease modeling, and personalized medicine research at unprecedented scale.

**Abbreviations:** ECG – Electrocardiogram, MRI – Magnetic Resonance Imaging, CT – Computed Tomography, CTCA – Computed Tomography Coronary Angiography, TEE – Transesophageal Echocardiography, LGE – Late Gadolinium Enhancement, GAN – Generative Adversarial Network, ScarGAN – Scar Generative Adversarial Network, STE – Speckle-Tracking Echocardiography, FFR – Fractional Flow Reserve, CRT – Cardiac Resynchronization Therapy.

***Supplementary Legend to Figure*** ***2****-* ***Applications of Variational Autoencoders (VAEs) in Cardiology.***

This legend expands upon the representative examples shown in **Figure 2** of the main manuscript, illustrating four major domains in which Variational Autoencoders (VAEs) are actively applied in cardiology.

**Top Left Panel**: This panel displays one electrocardiogram (ECG) waveforms generated by a Variational Autoencoder (VAE). The model was trained on real ECG signals to learn a compact latent representation capturing key temporal and morphological characteristics of cardiac electrical activity. Once trained, the VAE can sample from this latent space to generate new, physiologically realistic ECG waveforms. This application supports data augmentation in tasks such as arrhythmia classification, particularly where datasets are imbalanced or small. Synthetic ECGs produced in this manner reflect the natural variability of cardiac rhythms while preserving diagnostic integrity.

**Middle Left Panel**: This panel shows a hypothetical schematic of the reconstruction of three-dimensional cardiac anatomy from imaging data using a Variational Autoencoder (VAE).The model learns to encode high-dimensional cardiac imaging inputs,such as MRI or CT slices,into a lower-dimensional latent space that preserves essential structural features. From this compact representation, the VAE can decode and reconstruct detailed 3D heart geometries, including ventricular shape, myocardial contours, and chamber dimensions. This application enables efficient modeling of anatomical variability across patient populations and supports the identification of structural patterns associated with adverse outcomes, such as major adverse cardiac events (MACE). The panel illustrates how VAEs facilitate dimensionality reduction, anatomical encoding, and reconstruction in a clinically interpretable manner, contributing to risk stratification and computational phenotyping in cardiology.

**Top Right Panel**: This panel illustrates multimodal integration using a Variational Autoencoder (VAE), which simultaneously encodes cardiac MRI (structural data) and ECG (electrophysiological signals) into a unified latent space. The inclusion of MACE (Major Adverse Cardiac Events) reflects the use of this latent representation for outcome prediction, linking anatomical and electrical features to clinically meaningful endpoints.. The goal is to synthesize or analyze patient-level data that is both anatomically and electrically coherent. The inclusion of "MACE" (Major Adverse Cardiac Events) in the panel highlights the clinical application of this approach for risk prediction. By learning representations that span modalities, VAEs can support predictive modeling for adverse cardiovascular outcomes, enabling early identification of high-risk patients and enhancing decision support systems.

**Bottom Left Panel**: This panel depicts the application of Variational Autoencoders (VAEs) to model **ventricular motion** across the cardiac cycle. The image is a visual summary of learned ventricular motion, not a full cine loop, but a still frame representing how the VAE captures myocardial contraction patterns from the cardiac cycle. VAEs trained on cine MRI sequences can learn compact latent representations of temporal motion patterns, enabling the reconstruction or generation of realistic dynamic sequences. This allows for the study of myocardial mechanics in a fully unsupervised or semi-supervised manner. By encoding motion information into a latent space, VAEs facilitate detection of subtle wall motion abnormalities, support data-driven phenotyping of cardiomyopathies, and enable personalized modeling of cardiac function. The panel illustrates how VAEs extend beyond static image analysis to dynamic, time-resolved modeling of cardiac performance.

**Bottom Middle Panel**: This panel demonstrates the use of Variational Autoencoders (VAEs) for detecting myocardial ischemia on cardiac imaging, likely stress perfusion MRI or PET. The image shows a **conceptual ischemia map** generated through unsupervised detection using a Variational Autoencoder (VAE), where reconstruction errors or latent deviations highlight potentially ischemic myocardial regions.When the model is presented with ischemic or abnormal scans, it reconstructs them poorly in the affected regions, allowing for **unsupervised anomaly detection.** This reconstruction error can be mapped back to the image to **localize ischemic zones** without the need for pixel-wise manual labels. The panel reflects the utility of VAEs in identifying regional perfusion abnormalities and supporting diagnostic workflows where labeled data are limited or variable.

**Bottom Right Panel**: This panel illustrates the A schematic output of **unsupervised cardiac segmentation**, where a **Variational Autoencoder (VAE)** encodes anatomical structure and a **Generative Adversarial Network (GAN)** refines boundary precision. The panel symbolically shows how deep generative models can segment cardiac anatomy without relying on extensive manual annotations.In this approach, the VAE learns to organize anatomical variability in the latent space without requiring manual labels. Clusters in the latent space can correspond to different anatomical regions or pathological features. These embeddings can then guide segmentation or classification tasks through downstream clustering, anomaly detection, or semi-supervised learning.

**Abbreviations:** VAE – Variational Autoencoder, ECG – Electrocardiogram, MRI – Magnetic Resonance Imaging, MACE – Major Adverse Cardiac Events, PET – Positron Emission Tomography.

***Supplementary Legend to Figure 3.*** *-* ***Expanded Use Cases of Transformers in in Cardiology.***

This figure expands on **Figure 3** of the main manuscript, illustrating four primary domains in which Transformer-based architectures are being explored for cardiovascular applications. Each panel reflects an active research area where self-attention mechanisms have improved the interpretation of sequential, structured, and unstructured clinical data.

**Top Left Panel**: This panel illustrates the use of masked Transformer models for electrocardiogram (ECG) interpretation. The ECG waveform is visualized as a temporal sequence, with certain waveform segments shaded or marked as "masked." In this context, **"masked" refers to input regions that are intentionally hidden from the model during training,** forcing the model to learn contextual dependencies by predicting the missing values. Originally developed in natural language processing (e.g., BERT), masked Transformers adapt this strategy to time-series data, allowing the model to focus on **learning the global structure and temporal relationships** in ECG signals. During training, the model receives an incomplete ECG input and is optimized to reconstruct or classify it accurately based on surrounding unmasked data. This approach improves generalization, reduces overfitting, and enhances diagnostic performance,particularly when combined with convolutional layers for local feature extraction. The panel highlights the role of masked attention in improving computational efficiency while retaining the ability to detect arrhythmias and waveform abnormalities from limited or partially observed inputs.

**Top Right Panel**: This panel illustrates the use of Transformer-based language models, such as BERT (Bidirectional Encoder Representations from Transformers) and XLNet, for risk prediction in electronic health record (EHR) data. This panel illustrates the application of Transformer models such as BERT and XLNet for risk prediction using electronic health record (EHR) data. The visual includes symbolic representations of structured EHR elements (e.g., demographics, lab values) and unstructured text (e.g., clinical notes), highlighting the multimodal input these models can process. In this context, Transformers learn temporal and semantic relationships across diverse data types to predict clinical outcomes such as 6-month mortality or major adverse cardiac events (MACE). The panel emphasizes that these models are typically **pretrained on large corpora** and later **fine-tuned on task-specific cardiac datasets,** enabling them to extract clinically relevant features from both structured fields and free text. By applying self-attention mechanisms, BERT and XLNet capture complex interactions across time and variable types, outperforming traditional models in identifying high-risk patients. This panel showcases how language models originally developed for text analysis are now reshaping risk stratification and predictive analytics in cardiovascular medicine.

**Bottom Left Panel**: This panel illustrates the application of hierarchical Transformer models,specifically Hi-BEHRT (Hierarchical Bidirectional Encoder Representations from Transformers),to natural language processing (NLP) and longitudinal electronic health record (EHR) modeling. Patient data are shown as a temporal sequence of clinical events, such as diagnoses, prescriptions, lab results, and narrative notes, spanning multiple visits. Hi-BEHRT models this complex, multilevel structure by processing **individual clinical events within visits** (short-term context) and **temporal patterns across visits** (long-term context). By capturing both granular and temporal dependencies, it enables accurate modeling of disease progression, comorbidity interactions, and risk trajectories. The panel may also depict unstructured text input, highlighting the Transformer’s ability to extract predictive signals from free-text clinical notes. This architecture is particularly well suited for cardiovascular applications where long-range dependencies,such as the accumulation of risk factors,affect outcomes. The panel underscores how Transformers support interpretable, timeline-aware modeling for early identification of high-risk cardiac patients.

**Bottom Right Panel**: Bottom Right Panel: This panel illustrates the application of Transformer architectures to cardiovascular imaging analysis. On the left, a cardiac MRI cine frame shows a short-axis view of the left ventricle, representing standard input for structural and functional analysis. The center image, labeled “Amyloid” and “HCM,” depicts a Transformer-based classification output, highlighting the model’s ability to differentiate phenotypically similar cardiomyopathies,such as cardiac amyloidosis and hypertrophic cardiomyopathy,based on subtle imaging features. On the right, a fluoroscopic coronary angiogram is shown with a red-dotted overlay, suggesting the use of Transformers in dynamic catheterization lab imaging for tasks such as systolic wall motion tracking or stenosis detection. Together, these images reflect the versatility of Transformer models across modalities and tasks, including segmentation, phenotype classification, and procedural imaging interpretation

**Abbreviations:** ECG – Electrocardiogram, EHR – Electronic Health Record, BERT – Bidirectional Encoder Representations from Transformers, XLNet – Generalized Autoregressive Pretraining for Language Understanding, Hi-BEHRT – Hierarchical Bidirectional Encoder Representations from Transformers, HCM – Hypertrophic Cardiomyopathy .

***Supplementary Legend to Figure*** ***4****. -* ***Expanded Use Cases of Autoregressive Models in Cardiology.***

This legend expands on the content illustrated in **Figure 4**, which summarizes core applications of autoregressive (AR) modeling in cardiovascular signal analysis. Each panel demonstrates a distinct use case, ranging from ECG-based rhythm classification and heart rate variability (HRV) assessment to multivariate physiologic modeling and ICU forecasting. Together, these examples highlight the continued relevance of AR frameworks in contexts where interpretability, temporal structure, and physiological transparency are essential.

**Top Left Panel**: This panel schematically illustrates the application of autoregressive (AR) modeling to electrocardiogram (ECG) signals. The ECG waveform is shown as a time series input, with the accompanying label referencing the AR coefficients extracted from the signal. While not explicitly visualized, these coefficients are used in practice to characterize the signal’s temporal structure and support arrhythmia classification tasks. By analyzing segments of the ECG and estimating their AR coefficients, the model captures temporal dependencies and waveform morphology in a compact mathematical form. These coefficients serve as quantitative features that distinguish between normal sinus rhythm and arrhythmias (e.g., atrial fibrillation, premature ventricular contractions).

**Top Middle Panel**: This panel demonstrates the application of autoregressive (AR) models for spectral analysis of heart rate variability (HRV). The plot shows the power spectral density derived from RR interval time series, with clearly delineated low-frequency (LF) and high-frequency (HF) bands,used to quantify autonomic nervous system activity.AR spectral estimation methods model the time series as a stochastic process and estimate its spectrum through the parameters of the AR model, allowing for **high-resolution analysis even with short or noisy recordings**. Compared to traditional Fourier methods, AR-based approaches offer smoother spectral curves and better delineation of physiologic frequency bands. This panel underscores the importance of AR models in autonomic assessment and noninvasive monitoring of cardiovascular regulatory mechanisms.

**Top Right Panel**: This panel illustrates the application of **time-varying autoregressive (TVAR)** modeling to assess **heart rate variability (HRV)** under dynamic physiological conditions such as **rest and stress**. The two line plots represent RR interval time series extracted from ECG data, with visibly different patterns between resting and stressed states. At rest, HRV is typically higher and more structured, while under stress, the variability tends to decrease or shift in pattern due to altered autonomic balance. Unlike traditional frequency-domain methods such as the Fourier transform,which assume stationarity,TVAR models allow the AR coefficients to change over time, enabling real-time tracking of autonomic nervous system responses. This approach is especially valuable in scenarios like exercise, psychological stress testing, or perioperative monitoring. The panel conceptually demonstrates how TVAR modeling captures the **temporal evolution of autonomic function**, offering a physiologically grounded, high-resolution method for HRV analysis.

**Bottom Left Panel: This panel illustrates the application of autoregressive (AR) modeling to characterize cardiorespiratory interactions in patients with heart failure. The panel displays two synchronized physiological signals,typically respiratory effort and heart period (or RR interval),plotted over time. AR-based techniques, including bivariate and multivariate extensions, are used to quantify the directional influence between these signals, capturing respiratory–cardiac coupling and revealing the underlying autonomic regulation. In heart failure, impaired neural control and reduced vagal tone often lead to disrupted or attenuated coupling, which can be identified through changes in signal interdependence. By estimating the strength and temporal dynamics of these interactions, AR models provide a noninvasive assessment of autonomic function, offering valuable insight into disease progression and response to therapy in patients with chronic heart failure.**

**Bottom Middle Panel**: This panel illustrates the application of **autoregressive integrated moving average (ARIMA)** models to forecast changes in vital signs within a **critical care setting,** such as the **intensive care unit (ICU).** The plot shows actual versus predicted values for a physiological variable,commonly **heart rate, blood pressure,** or **oxygen saturation**,monitored over time following cardiac surgery or during acute clinical episodes. ARIMA models extend standard AR models by incorporating **differencing** (to account for non-stationary trends) and **moving average terms** (to model noise and short-term fluctuations), resulting in robust short-term forecasting capabilities in highly dynamic environments. These models have been shown to **outperform traditional linear regression** in detecting early signs of physiological instability, making them valuable components of **early warning systems**. The panel demonstrates how transparent and well-established statistical modeling frameworks can be effectively applied to **support real-time clinical decision-making in acute cardiovascular care.**

**Bottom Right Panel**: **Bottom Right Panel**: This panel conceptually represents the use of **multivariate autoregressive (MVAR)** modeling to estimate **baroreflex sensitivity (BRS)**, a key indicator of autonomic cardiovascular regulation. Although the visual is schematic and lacks explicit signal traces, it refers to the simultaneous modeling of **systolic blood pressure** and **RR intervals** to capture **directional physiological interactions.** MVAR models estimate both feedforward (blood pressure to heart rate) and feedback (heart rate to blood pressure) influences, providing a more physiologically realistic assessment of cardiovascular control than static regression approaches. In practice, BRS is quantified as the gain of RR interval response to pressure fluctuations (expressed in ms/mmHg). While the figure is abstract, it points to the role of MVAR in **noninvasive, dynamic analysis of autonomic function,** relevant to risk stratification in conditions such as hypertension, syncope, and heart failure.

**Abbreviations:** AR – Autoregressive, HRV – Heart Rate Variability, ARIMA – Autoregressive Integrated Moving Average, MVAR – Multivariate Autoregressive, BRS – Baroreflex Sensitivity, ECG – Electrocardiogram, ICU – Intensive Care Unit.

***Supplementary Legend to Figure*** ***5****. -* ***Expanded Use Cases of Diffusion Models in Cardiology.***

This legend expands on the content illustrated in **Figure 5**, which highlights key applications of diffusion models in cardiology. Each panel showcases a distinct use case,ranging from synthetic image and signal generation to physiologic reconstruction and simulation,demonstrating the versatility of diffusion-based frameworks in data augmentation, privacy-preserving synthesis, and virtual modeling.

**Top Left Panel**: This panel illustrates the use of diffusion models for generating **synthetic cardiac magnetic resonance imaging (MRI)** data. The visual shows two axial slices of cardiac MRI, representing either paired real–synthetic images or two independently generated synthetic outputs. Diffusion models are trained to reverse a noise-adding process, learning to progressively denoise random inputs into anatomically realistic outputs. When applied to cardiac MRI, these models capture fine-grained spatial features, including myocardial contours, ventricular geometry, and chamber orientation, while preserving the structural integrity of real clinical images. This technique enables the generation of **privacy-compliant synthetic datasets,** useful for model training, testing, or augmentation in settings with limited annotated data or strict data-sharing constraints. The panel highlights the **high structural fidelity** of diffusion-generated cardiac MRIs and their potential role in **data augmentation and anonymized imaging simulation.**

**Top Right Panel**: This panel symbolically represents the use of **denoising diffusion probabilistic models (DDPMs)** to generate synthetic electrocardiogram (ECG) signals. The displayed waveform is a clean, structured ECG trace, serving as an example of the type of output diffusion models can produce. These models reconstruct signals by reversing a stepwise noise-adding process, enabling the generation of physiologically realistic waveforms that capture key features such as P waves, QRS complexes, and T-wave morphology. While the panel shows a single illustrative trace, it highlights the broader application of diffusion models for signal synthesis, particularly in augmenting underrepresented arrhythmic classes and supporting diagnostic algorithm development.

**Bottom Left Panel**: This panel illustrates the use of diffusion models for reconstructing **three-dimensional cardiac activation maps** from sparse or incomplete imaging data. The colorful volumetric shape represents electrical activation across the myocardium, typically derived from modalities such as ECG imaging (ECGi) or tagged MRI. Diffusion-based frameworks can be trained to infer full 3D activation patterns from limited 2D slices or surface maps, progressively denoising partial inputs into physiologically plausible 3D reconstructions. This method enables **noninvasive characterization of electromechanical delay,** which is critical for diagnosing conduction abnormalities and planning interventions such as cardiac resynchronization therapy (CRT).

**Bottom Right Panel: This panel symbolically represents the emerging use of diffusion models to simulate cardiac excitation wavefronts,the electrical signals that coordinate myocardial contraction. The stylized heart with radiating concentric patterns evokes the propagation of excitation across the myocardium. While not depicting a true simulation output, the image illustrates the conceptual shift from traditional physics-based electrophysiological models toward data-driven generative approaches. Diffusion models are being explored as scalable tools for virtual testing, arrhythmia mechanism modeling, and patient-specific simulation in cardiac electrophysiology.**

**Abbreviations:** MRI – Magnetic Resonance Imaging, ECG – Electrocardiogram, CRT – Cardiac Resynchronization Therapy, DDPM – Denoising Diffusion Probabilistic Model.

***Supplementary Legend to Figure*** ***6****. -* ***Expanded Use Cases of Digital Twins and Synthetic Cohort Simulators in Cardiology.***

This legend expands on **Figure 6**, which contrasts the roles of digital twins and synthetic cohort simulators in data-driven cardiology. The figure illustrates how digital twins enable personalized, dynamic modeling of cardiac structure and function, while synthetic cohorts support population-level simulations, virtual trials, and privacy-preserving AI development.

**Left Panel**: This panel symbolically represents a **patient-specific digital twin** of the heart used for **simulation and procedural planning.** The image shows a patient connected to an ECG monitor with a stylized heart illustration superimposed on the chest, representing the concept of personalized cardiac modeling. Digital twins integrate multimodal patient data,such as cardiac imaging, ECG, and anatomical information,to create individualized simulations reflecting the patient’s current state. These simulations are being explored for use in **arrhythmia mapping, therapy response prediction,** and **interventional strategy rehearsal**. Although simplified, the panel highlights the conceptual role of digital twins in advancing precision cardiology through **interpretable and physiologically grounded virtual modeling.**

**Middle Panel**: This panel illustrates the use of **synthetic ECGs** and **risk modeling tools** derived from simulated patient data. The visual includes ECG waveforms, a human figure with cardiovascular symbolism, and a risk-related bar chart,together symbolizing the integration of simulated signals and machine learning–based risk stratification. These synthetic signals are typically generated using models trained on large clinical datasets and then anonymized to preserve privacy. Once generated, synthetic ECGs can be used to test risk prediction pipelines, validate algorithms, and train models under diverse conditions. This approach supports privacy-compliant, reproducible development of diagnostic tools, bridging individualized simulation (digital twins) and population-scale modeling (synthetic cohorts).

**Right Panel**: This panel symbolically represents a **synthetic cohort simulator,** used to generate large-scale virtual patient populations for **in silico studies** and **virtual anatomical trials**. The visual shows a series of uniform human silhouettes with heart icons, reflecting the creation of standardized yet statistically diverse virtual subjects. These simulators are trained on real-world data and reproduce the statistical features of actual populations,including distributions of age, comorbidities, ECG findings, and anatomical characteristics,without exposing identifiable information. Such synthetic cohorts enable robust validation of medical devices, risk models, and treatment strategies, particularly in settings with ethical or regulatory constraints. The panel highlights the role of synthetic populations in **scalable, privacy-preserving modeling** for cardiovascular research and innovation.

**Abbreviations:** ECG – Electrocardiogram, CRT – Cardiac Resynchronization Therapy, AI – Artificial Intelligence, XR – Extended Reality.
