# Supplementary figures and images for: Synthetic artificial intelligence in cardiology: from generative models to clinical applications
Source: Eur Heart J Open. 2026 Mar 1;6(2):oeag026. doi: 10.1093/ehjopen/oeag026 (PMC13070426; doi:10.1093/ehjopen/oeag026)

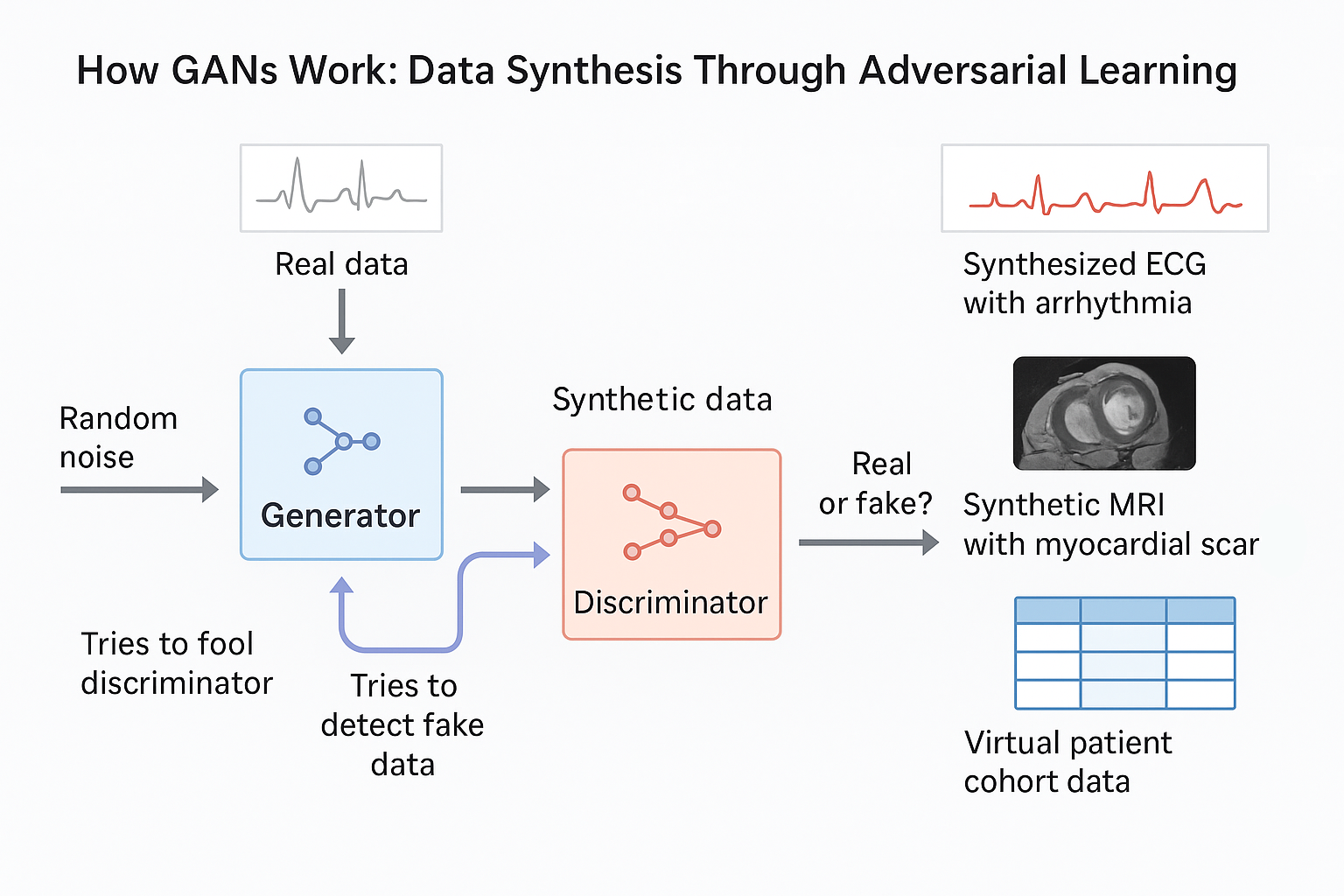

Supplement: oeag026_Supplementary_Data [file oeag026_supplementary_data.zip › Figure S1.tif]

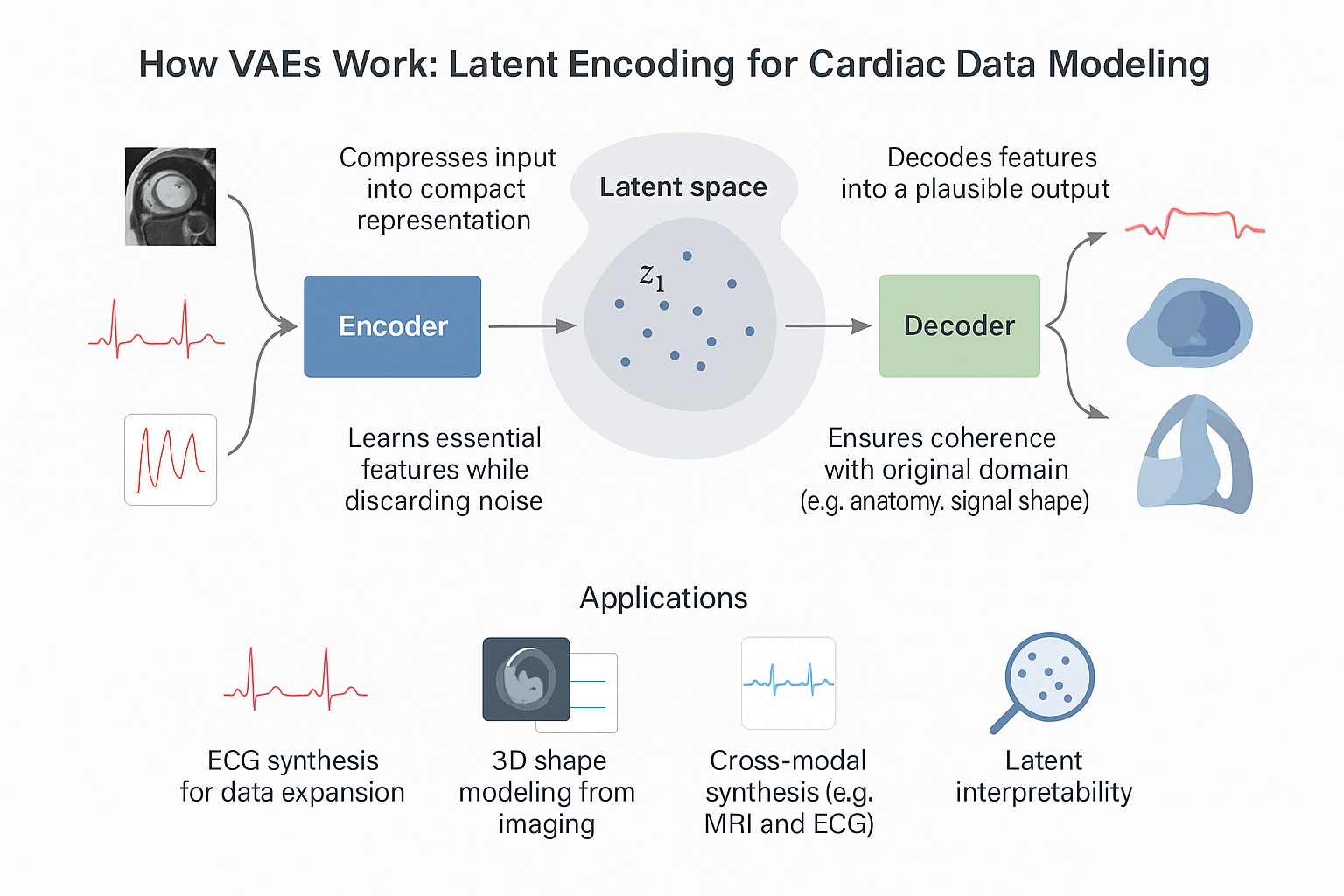

Supplement: oeag026_Supplementary_Data [file oeag026_supplementary_data.zip › Figure S2.tif]

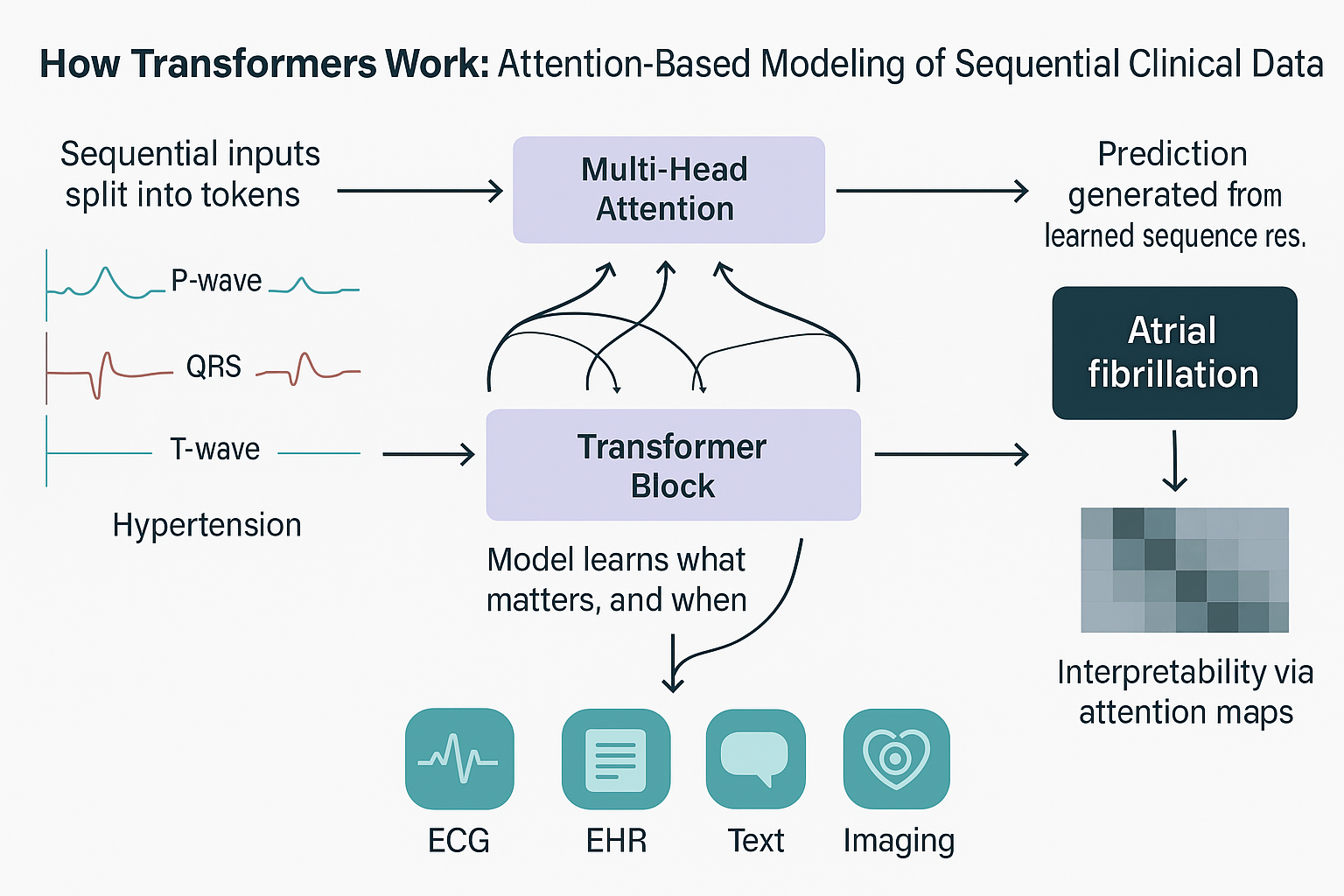

Supplement: oeag026_Supplementary_Data [file oeag026_supplementary_data.zip › Figure S3.tif]

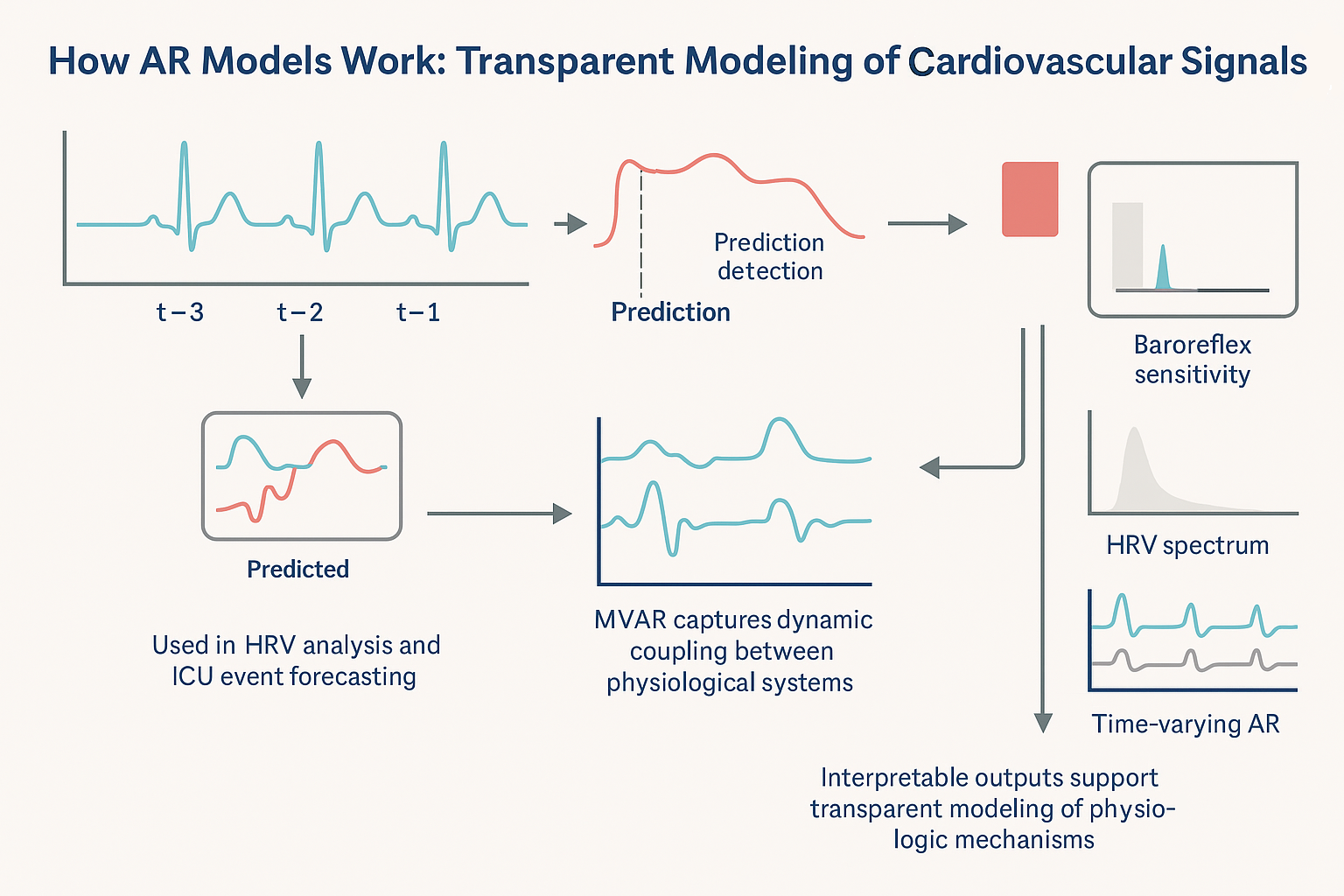

Supplement: oeag026_Supplementary_Data [file oeag026_supplementary_data.zip › Figure S4.tif]

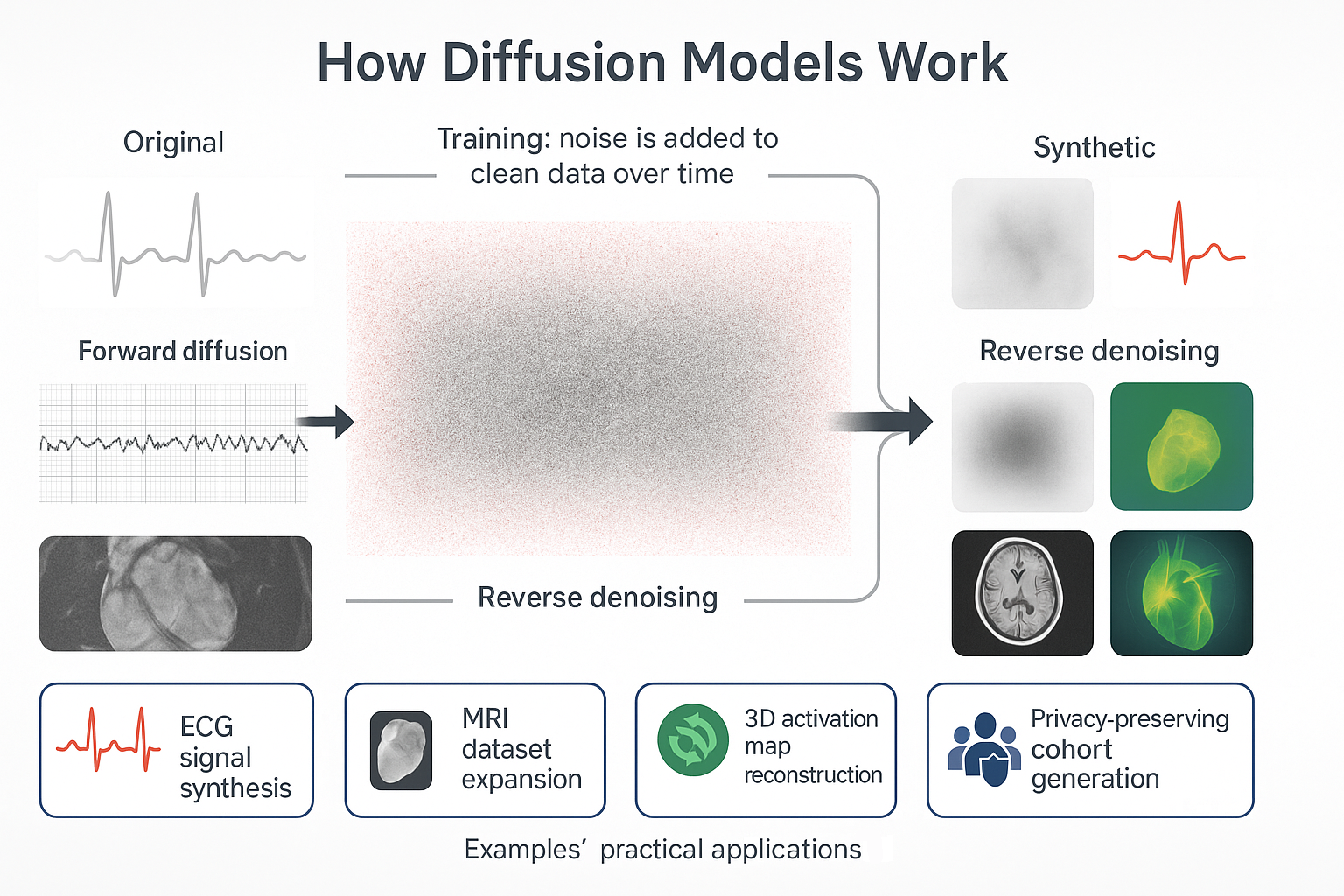

Supplement: oeag026_Supplementary_Data [file oeag026_supplementary_data.zip › Figure S5.tif]

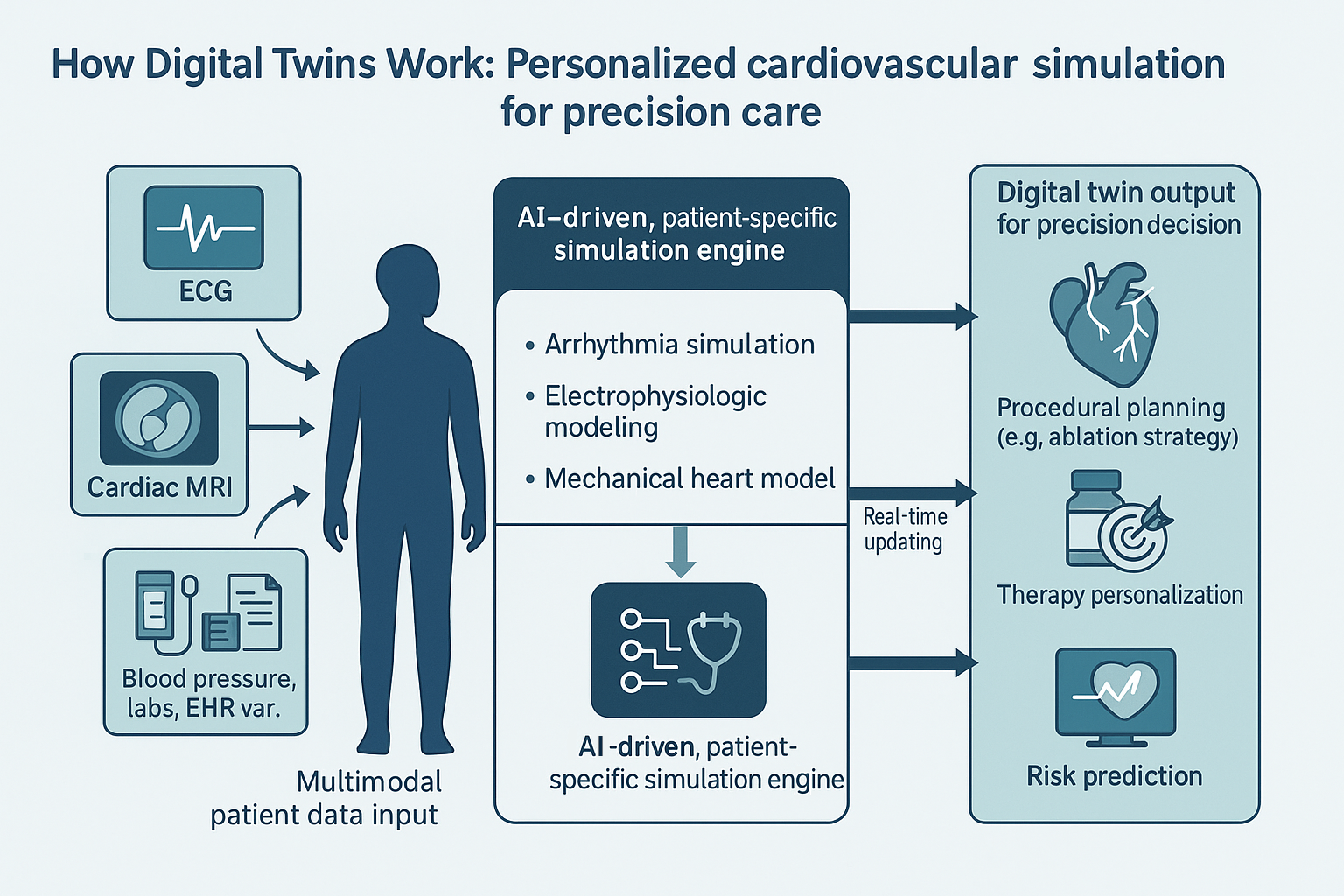

Supplement: oeag026_Supplementary_Data [file oeag026_supplementary_data.zip › Figure S6.tif]

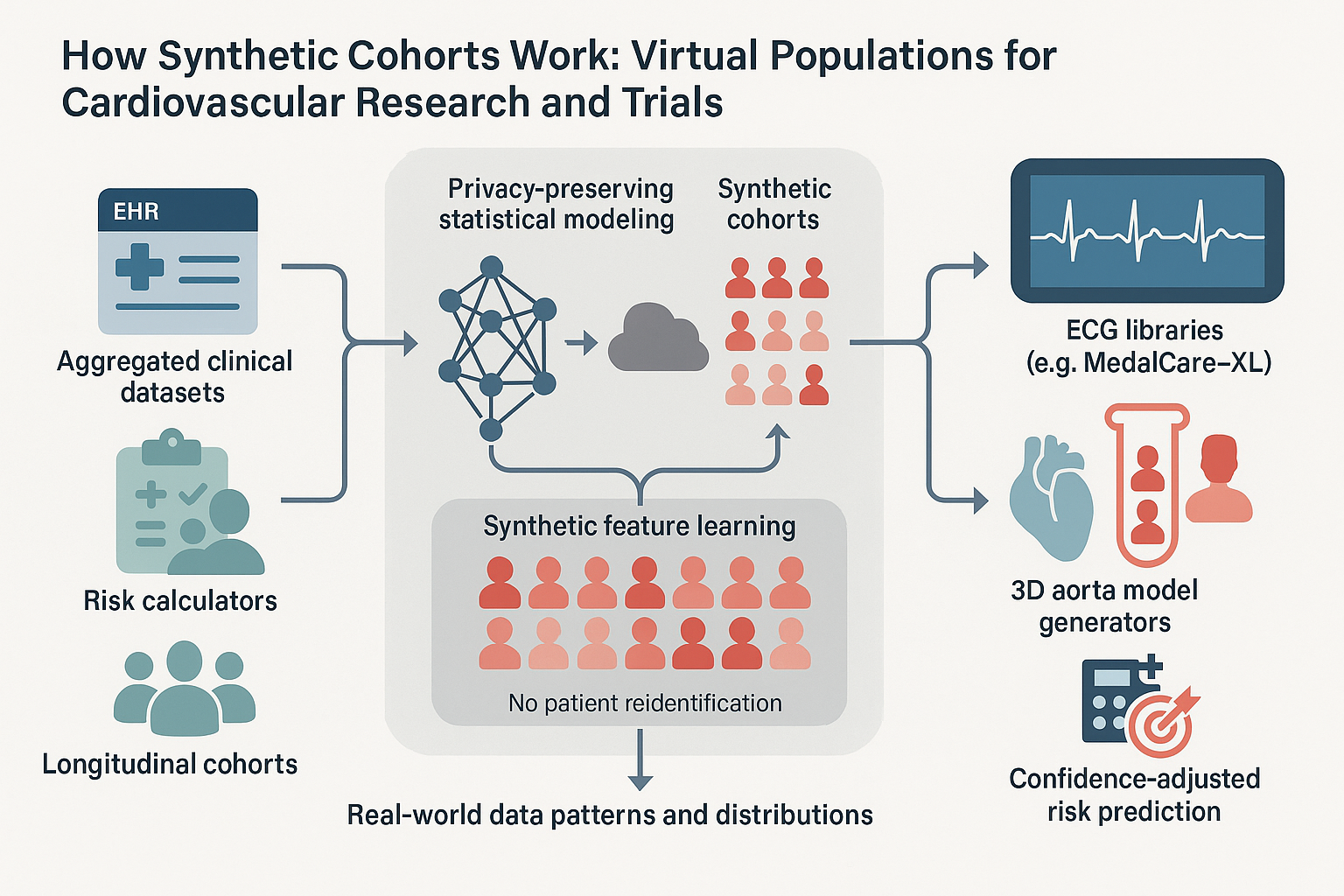

Supplement: oeag026_Supplementary_Data [file oeag026_supplementary_data.zip › Figure S7.tif]
